# Scalable, modular continuous wave functional near-infrared spectroscopy system (Spotlight)

**DOI:** 10.1117/1.JBO.28.6.065003

**Published:** 2023-06-13

**Authors:** Daniel Anaya, Gautam Batra, Peter Bracewell, Ryan Catoen, Dev Chakraborty, Mark Chevillet, Pradeep Damodara, Alvin Dominguez, Laurence Emms, Zifan Jiang, Ealgoo Kim, Keith Klumb, Frances Lau, Rosemary Le, Jamie Li, Brett Mateo, Laura Matloff, Asha Mehta, Emily M. Mugler, Akansh Murthy, Sho Nakagome, Ryan Orendorff, E-Fann Saung, Roland Schwarz, Ruben Sethi, Rudy Sevile, Ajay Srivastava, John Sundberg, Ying Yang, Allen Yin

**Affiliations:** Meta Platforms, Inc., Menlo Park, California, United States

**Keywords:** functional near-infrared spectroscopy, brain–computer interface, diffuse optical tomography

## Abstract

**Significance:**

We present a fiberless, portable, and modular continuous wave-functional near-infrared spectroscopy system, Spotlight, consisting of multiple palm-sized modules—each containing high-density light-emitting diode and silicon photomultiplier detector arrays embedded in a flexible membrane that facilitates optode coupling to scalp curvature.

**Aim:**

Spotlight’s goal is to be a more portable, accessible, and powerful functional near-infrared spectroscopy (fNIRS) device for neuroscience and brain–computer interface (BCI) applications. We hope that the Spotlight designs we share here can spur more advances in fNIRS technology and better enable future non-invasive neuroscience and BCI research.

**Approach:**

We report sensor characteristics in system validation on phantoms and motor cortical hemodynamic responses in a human finger-tapping experiment, where subjects wore custom 3D-printed caps with two sensor modules.

**Results:**

The task conditions can be decoded offline with a median accuracy of 69.6%, reaching 94.7% for the best subject, and at a comparable accuracy in real time for a subset of subjects. We quantified how well the custom caps fitted to each subject and observed that better fit leads to more observed task-dependent hemodynamic response and better decoding accuracy.

**Conclusions:**

The advances presented here should serve to make fNIRS more accessible for BCI applications.

## 1 Introduction

Continuous-wave functional near-infrared spectroscopy (CW-fNIRS) can be used to non-invasively measure cortical hemodynamics^1^ by emitting near-infrared light into the brain at the scalp and sensing the portion that returns to the scalp after propagating diffusely through cortical tissue. A local increase in cortical neural activities changes the surrounding tissue’s blood oxygenation level via neurovascular coupling,^2^ known as the hemodynamic response. The change in the blood’s oxygenation level modulates the amount of infrared light that can propagate through and subsequently be detected by optical sensors at the scalp. The more prominent neuroimaging approach in humans, blood oxygenation level-dependent (BOLD) functional magnetic resonance imaging (fMRI),^3^ also measures the hemodynamic response and has seen wide applications in cognitive neuroscience,^4^ translational medicine, and clinical practice.^5^ Compared with fMRI, functional near-infrared spectroscopy (fNIRS) has higher portability and tolerance for motion, higher to comparable temporal resolution, but less spatial resolution, depth of view, and signal-to-noise ratio (SNR).^6^^,^^7^ As a result of its relative advantages, the fNIRS field has grown rapidly into many cognitive neuroscience and translational medicine research areas^8^^,^^9^ in the past few decades. In the recent years, fNIRS has also been used to build non-invasive brain–computer interface (BCI)^10^^,^^11^ communication systems that allow the use of brain activity to control computers or other external actuators,^12^ which has potential applications in neurophysiology, neurorehabilitation, and even consumer products,^13^^–^^15^ due to its non-invasive nature and potential portability.

Traditional CW-fNIRS imaging uses sparse arrangements of NIR source–detector (SD) measurements, resulting in significantly lower spatial resolution than fMRI.^16^ Recent developments in diffuse optical tomography (DOT)^17^^–^^19^ and high-density DOT (HD-DOT),^20^^,^^21^ which use increasingly higher numbers of NIR light sources and detectors to provide overlapping spatial sampling of the target object, have improved the spatial resolution of the modality dramatically, enabling three-dimensional, high-resolution functional neuroimaging with wide field-of-view.^17^^–^^21^ Increasing the density of the source and detector (optode) arrays provides a number of benefits, including greater lateral image resolution,^22^ higher SNR,^23^ improved depth sensitivity and specificity,^24^ and easier removal of physiological confounds.^25^^–^^27^ The initial advancements in HD-DOT’s optode density were driven by coupling more optical fibers to the subject’s scalp, approaching ∼200 in some cases,^20^ reducing the technique’s portability and ease of use—key advantages that initially drove the growth of the fNIRS field. More recent advances in fiberless CW-fNIRS technology have resulted in several commercial devices such as NIRx’s NirSport 2^28^ and Gowerlab’s Lumo^29^ that achieved high optode numbers and density without compromising portability and ease of use. However, both devices still lag behind the current HD-DOT state-of-the-art^30^ in either optode density or SNR.

We present a CW-fNIRS system, “Spotlight,” composed of compact modular arrays of 39 detectors and 41 dual-wavelength NIR light-emitting diode (LED) sources (680 and 850 nm), with 6.5 mm interoptode spacing. Each module is roughly palm-sized (∼88  mm diameter), and multiple modules can be installed on a compatible head cap to increase field-of-view. Our system achieves optode number, density, and SNR similar to current state-of-the-art in HD-DOT^28^ in an easy-to-use portable form factor by combining advances in silicon photomultiplier (SiPM) detectors,^31^ flexible printed circuit board (FPCB) fabrication, optical ferrule design, customized 3D cap printing, and modular design approaches.

We validated Spotlight in both *in vitro* experiments using custom phantoms with optical properties similar to the human head and *in vivo* human subject experiments. Human experiment subjects performed short-duration (3 and 6 s) finger-tapping tasks with either the left or right hand. Hemodynamic response was measured bilaterally in the motor cortex, with activity patterns consistent with contralateral motor control and utilized to successfully decode the tasks’ laterality conditions. Finally, analysis was performed on factors contributing to subject variability in signal quality and decoding accuracy to inform future CW-fNIRS designs.

## 2 Materials and Methods

### 2.1 System Overview

**Fig. 1:**
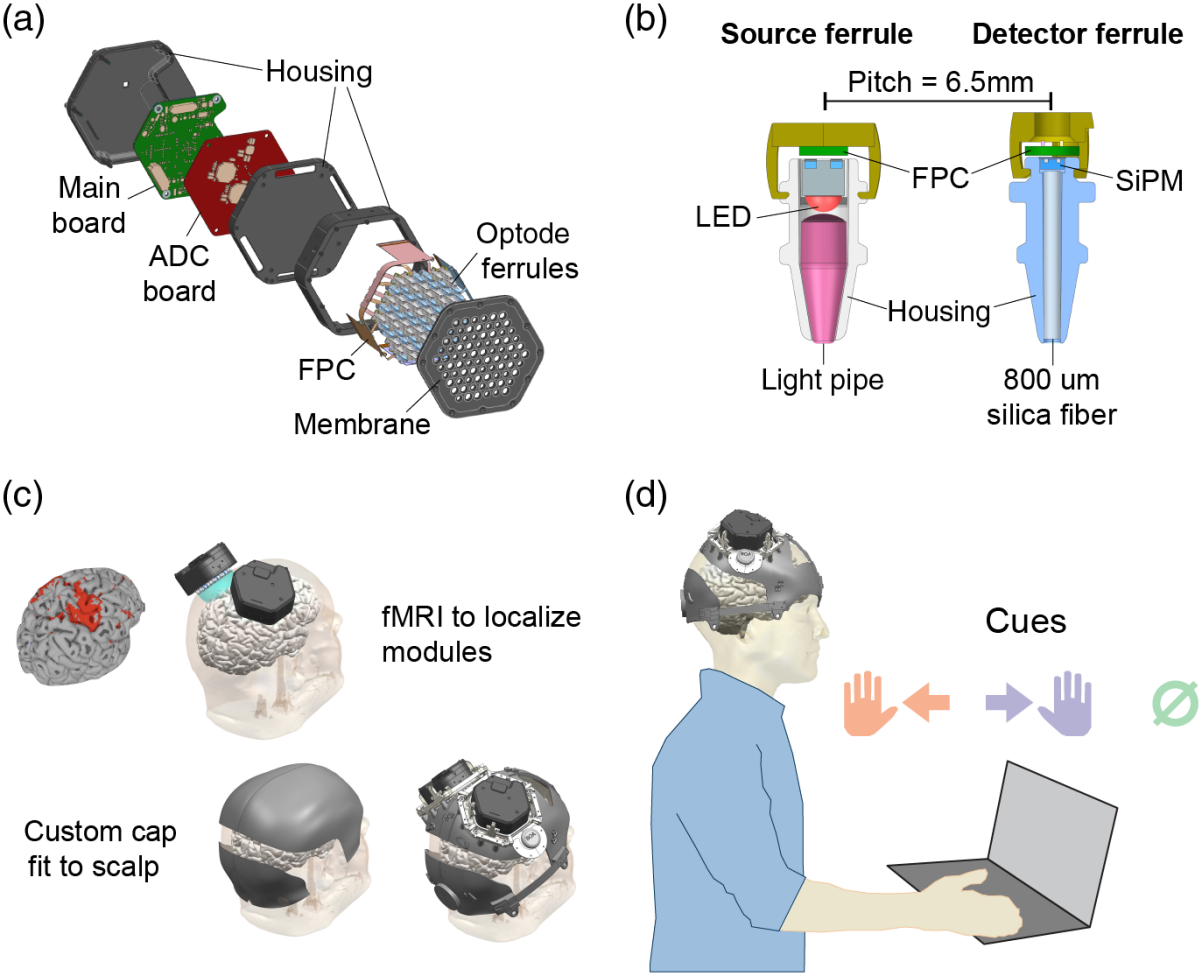
Modular CW-fNIRS system overview: (a) each module packages a control and an ADC board to drive and sample from source and detector optodes embedded in a flexible membrane. (b) Optical ferrules were designed to comb through hair, enable high-density optode placements, and tight optical coupling to and from the scalp. (c) A custom personalized self-donnable cap designed from MRI data for one subject. Modules are aligned over the centroid of the hand-motor activation areas, and cap shell shape derived from scalp surface and landmarks enable consistent placement of the cap. (d) Illustration of the human-subject finger-tapping protocol. Participants are cued to tap their left fingers, right fingers, or stay still (in the “null” condition).

The full system used for *in vivo* validation consists of two independent optical sensor (optode) modules [Fig. 1(a)] mounted on a custom self-donnable cap [Fig. 1(c)], over the hand-motor cortical areas. Each module has dimensions 84.5×82×38.4  mm, weighs 163 g, and contains 41 source and 39 detector optodes [Fig. 1(b)]. Each source optode contains LEDs emitting light of two wavelengths, 850 and 680 nm, and each detector optode contains an SiPM light detector (OnSemi MicroRD 10035). In one module, 41 sources coupled with 39 detectors with 2 wavelengths, there are 3198 channels. Small optomechanical structures called optical ferrules were developed [Fig. 1(b) and Sec. 1.1 in the Supplementary Material] to guide light from LEDs to the scalp and from the scalp to SiPM detectors. These ferrules can comb through hair and enable dense optode packing, with a minimum SD optode separation of 6.5 mm. The optodes are embedded in a flexible silicone membrane (see Sec. 1.6 in the Supplementary Material) enclosed by the module housing, such that when the module is pressed against the scalp, the optodes can make contact following its curvature. Within the module housing, the optodes are mounted on flexible FPCBs (see Sec. 2.1 and Fig. S1A in the Supplementary Material) with tree-branch-like patterns to provide compliance with the flexible membrane.

The coverage area of one module is 62 mm in diameter and can be extended by placing multiple modules side by side, while still maintaining the advantage of high optode density. This provides substantial advantages over conventional high-density fNIRS systems, in which scaling up the number of optodes and coverage area can be more demanding. However, the gap between the modules where optodes cannot be placed creates areas where sensitivity is lower than the center of the modules. We minimized this non-ideal gap to 25 mm. Modules are designed to conform to the curvature of the scalp with a tensioned elastic membrane that is held taut by the edge of the module, resulting in a 12.5 mm border. To ameliorate the concern of an overly large gap between adjacent modules, we use localization methods (Sec. 8 in the Supplementary Material) to center the modules over the desired regions.

The module mounting caps can be customized to accommodate different numbers and locations of optode modules. The mounting caps used in the human experiments were either personalized caps designed from structural MRI scans [Fig. 1(c)] or nearest-neighbor caps. Personalized caps were designed from structural MRI scans of the participants such that the optode modules are aligned over the centroid of hand motor activations, and the cap shell shape aligns to the scalp surface and landmarks to enable consistent cap fit. Participants without structural MRI scans used nearest-neighbor caps, which were available personalized caps with the closest size match.

### 2.2 Module Operation

Each module contains an analog digital converter (ADC) board and a mainboard for signal acquisition, data processing and transfer [Fig. 1(a) and also see Sec. 2.9 in the Supplementary Material]. The mainboard contains a FPGA (Xilinx XC7S50) and a microcontroller (STEMicroelectronics STM32F765NIH6), which collectively interfaces with multiple digital analog converters (DACs), ADCs, and sensors for driving the LEDs and reading from the SiPMs. The mainboard provides communication with a host-computer either directly or by connecting into our custom USB hub (spider and see Sec. 3.3 in the Supplementary Material), both through USB2.0.

Up to six Spotlight modules can connect via type-C USB cables to spider, which provides pulse-per-second synchronization signal and debugging signals to the modules (see Sec. 2.12 in the Supplementary Material). The hub also has additional analog/digital inputs for connecting peripheral devices. The hub interfaces bidirectionally with the host PC through a single Type-C USB2.0 cable (see Sec. 3.3 in the Supplementary Material).

During signal acquisition, the source LEDs are multiplexed to enable acquisition from all the SD pairs without interference. The standard illumination pattern involves a 1 ms pulse width alternating between 680 and 850 nm wavelengths on the same LED. Between each pulse is a 1 ms ambient frame, during which the signal is used to measure ambient light levels. Each module has a system effective duty cycle of 50%, 164 ms cycle time, and 6.1 Hz system frequency (see Sec. 2.4 in the Supplementary Material).

### 2.3 Custom Cap Design

The modules are fixed on the head over the motor area for finger tapping using custom-designed caps. Personalized caps are designed from structural MRI scans of subjects referencing the 3D scalp surfaces and localized over the centroid of hand motor activation [Fig. 1(c)]. The cap shell shapes were derived from the structural MRI scalp surface of the subject and aligned to the landmarks of the skull: the inion, nasion, and left and right auricular points. These landmark alignments ensured that the cap would be aligned consistently each time the cap is donned. Module mounting locations were centered over the hand motor cortex as determined from MRI data. Modules were mounted using an adjustable ratcheting spring mount that pressed the optodes against the scalp. The flexibility of the elastic springs enabled motion to comb optodes through hair while being self-centering.

To expand the subject pool, those who did not have MRI scans were matched to a “nearest neighbor” cap with the closest size match based on measurements of the inion to nasion distance and the distance between the left and right auricular points over the top of the head. Five participants used fMRI-personalized custom caps (EBS159, CRP353, 179 MST704, UYH816, and PJY122), whereas the other five used nearest-neighbor custom caps. Of those with personalized caps, the motor area module placements were identified via functional fMRI experiments for three participants, and via structural fMRI data for the other two participants. To evaluate the effect of variability in module placement for subjects without fMRI, adjustable caps (Fig. S25 in the Supplementary Material) were designed for two participants to further understand the effect of modules placed off-center from the active finger-tapping region (see Sec. 8 in the Supplementary Material).

### 2.4 Optical Efficiency Measurement

The light intensity of the optical sources was validated using a custom light integrating sphere (Labsphere P/N CSTM-LPMS-060-SL) with a large enough input port (2.75 in.) to accommodate an entire Spotlight module. The measurements near the center of the module tend to be more accurate, while LEDs near the edge will incur ∼5% variations. The LED drive current was set continuously to 250 mA for all sources during the validation experiments (see Sec. 2.3 in the Supplementary Material).

The SiPM detectors were measured using the same custom integrating sphere from Labsphere (P/N CSTM-LPMS-060-SL) with a large enough input port (2.75 in.) to accommodate either a single module for SiPM with ferrules or a SiPM FPC fixture for SiPM without ferrules. To derive detector efficiency, the 680 nm (Thorlabs) laser injects light into the sphere with a known intensity, and the voltages measured from SiPM with ferrules are compared against those from SiPMs without ferrules.

### 2.5 Static Phantom

Static homogeneous phantoms were used to conduct *in vitro* system benchmarking consistently. The core of the phantom is a custom mixed silicone made of PDMS Sylgard 182 and a mixture of titanium dioxide and black manganese-based pigment made by Douglas and Sturgees.^32^ Ratios were adjusted to achieve optical properties close to that of human tissue ($\mu_{a}=0.02\;{\mathrm{mm}}^{-1}$ and $\mu_{s^{\prime }}=1\;{\mathrm{mm}}^{-1}$, based on the previous experiments^33^). Silicone was used over harder polyurethane mixtures to emulate the softness of the scalp. The softness allows the ferrules to “dig” into the surface and reduces the potential for excessive photons passing from LEDs directly to the detector. The final assembly is 110 mm in diameter and 40 mm thick (Fig. S11 in the Supplementary Material).

### 2.6 System Benchmarking

We measured and tuned the performance of the sensor modules on phantoms before conducting the human validation studies, such that the effects of cap fitting and individual physiological differences were minimized. The module was placed on top of the static phantom during these measurements. The sensor tuning process aimed to find the optimal device parameters to balance the tradeoff between maximizing the detector power and minimizing the sensor noise and signal drift.

The Spotlight modules were evaluated with the following metrics to compare individual module performance, and a particular module’s *in vitro* and *in vivo* performances. The measured signal (W) was preprocessed with a fifth-order Butterworth 0.01 Hz high-pass filter to remove slow thermal drift prior to the calculations.

1. Median detector power (W) was calculated as the median of temporal mean power detected by channels with SD distance in the range [27, 33] mm. Because the measured power decreases exponentially with SD-distance, we limited this metric’s calculation to a specific SD-range for simplicity. The distance range was chosen as prior literature suggests that 30 mm SD separation provides good cortical sensitivity and is often employed in fNIRS studies.^24^^,^^26^
2. Median detector noise density (W/rtHz) of a module was calculated from channels with SD-distance >55  mm, as the light attenuation at these distances are such that the detectors’ measurements are dominated by detector noise. System-level noise-equivalent power (NEP) density for the selected SD pairs was calculated as the standard deviation of the temporal mean of 1 s rolling windows. The median of these NEPs was taken to be the final metric for a module. This metric can be thought of as the noise density at 1 Hz and has the units W/rtHz. Note that this noise figure is different from the device-level NEP commonly presented in avalanche photodiodes (APD)-based fNIRS devices,^34^ taking into account noise effects from the SiPM device, optode assembly, and system operation.
3. *Percent of good channels (%)*. SNR for individual channels was calculated as ratios between mean power and detector noise density. Channels with SNR>100 were deemed “good.” The final metric here is taken to be the percentage of good channels with SD separation in the range [27, 33] mm.

Note that as detected power of a channel decreases with increasing SD-distance due to scattering attenuation, the SNR and detector power metrics presented above are specified within the representative range of [27, 33] mm.

As the operating characteristics of the LED sources and SiPM detectors change with temperature, measurements were taken only after the module had reached thermal stability after being on for at least 10 min (see also Sec. 2.8 in the Supplementary Material). The calculations were then performed on the subsequent 30 min of data in both phantom and *in vivo* experiments.

Spotlight’s system dynamic range was calculated from the light fall-off curve^20^ collected from static phantom measurements (Fig. S13 in the Supplementary Material), taking the maximum measured power range (see Sec. 2.7 in the Supplementary Material).

### 2.7 Experiment Control

A custom software framework, Labgraph (see Sec. 9 in the Supplementary Material), was used during all human subject experiments to stream data from Spotlight, preprocess data, control experiment flow, present protocol stimulus, log keyboard events, and present visual feedback.

### 2.8 Human Data Collection: IRB and Experimental Design Ethics

The experiments were conducted following authorization from Meta Reality Lab’s Research Committee, which includes reviews for environmental health and safety, optical safety, and hardware safety. The experiments were also approved by an external Institutional Review Board (IRB) in accordance with the Nuremberg Code, Declaration of Helsinki, and the Belmont Report.

Data were collected from ten participants: three female and seven male, with an average age of 33.2 years. Nine subjects are right-hand dominant, and one subject is left-hand dominant. No subject had a known history of any neurologic or psychiatric disorder. All participants received and signed an informed consent document containing information about experiment participation, compensation, risks, and benefits, in addition to sections informing subjects of data usage, rights during the experiment, and data/confidentiality protections. Subjects also met with a research assistant who solicited and answered questions, confirmed understanding, and evaluated comfort. Randomized alphanumeric IDs were generated to protect anonymity of participants.

### 2.9 Experiment Protocol (“Two Duration Finger Tapping Protocol”)

All participants participated in the hemispheric laterality experiment described below.

**Fig. 2:**
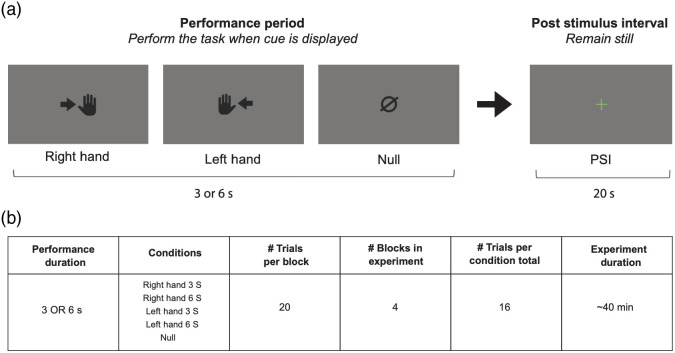
Experimental design of the hemispheric laterality experiment. (a) Illustration of the trial structure: the performance period consisted of right-hand tapping, left-hand tapping, or null (no tapping), for a duration of 3 or 6 s. The PSI period was 20 s. (b) Summary of the experiment: 20 trials were collected per block and 4 blocks were collected total, for a total of 16 trials per condition for the whole experiment. The experiment lasted roughly 40 min.

The hemispheric laterality experiment was modeled after Birn et al.^35^ to replicate established results in the literature. Two 40-min sessions were collected on two separate days (Fig. 2). When the stimulus displayed a hand cue, subjects tapped their thumb, index, and little finger sequentially until the cue disappeared (for 3 or 6 s). Participants remained at rest during the null condition and also during the post-stimulus interval (PSI) period (20 s in duration). There were five conditions in total: right-hand 3 s, right hand 6 s, left hand 3 s, left hand 6 s, and null. Consecutive null trials were not allowed (to allow for a more engaging experiment). Twenty trials (four trials per condition) were grouped into a “block” where the subjects continuously performed the task. Each block was preceded and followed by 20 additional seconds of rest, and self-paced rest time was allowed between blocks. In each session, four blocks of data were collected in total, resulting in 80 trials (16 trials per condition).

During experiment sessions, subjects were seated in front of a monitor with both hands on the keyboard. The displayed instructions before the start of each experiment reminded subjects: (1) to stay still, (2) to tap their fingers in the correct order, (3) to attend to each finger as it was being tapped (4) that there would be a break following each block. Breaks were self-paced; subjects would advance onto the next block by pressing the spacebar. To the best of their ability, subjects conducted experiments in the same lighting conditions. All subjects underwent a cap-fitting procedure before the protocols started to maximize optode SNR while maintaining comfort. The SNRs were evaluated in real time and visualized with a light-fall off curve, along with a dynamic visualization superimposing the mean power of each detector on the module layout.

Additional experiments were conducted on a subset of participants. The real-time closed-loop hemispheric laterality experiment was conducted to evaluate the effects of real-time closed loop feedback on decoder performance. The digit-localization experiment (see Sec. 7 in the Supplementary Material) was conducted to further evaluate the ability to discriminate between individual digits within a single hemisphere/optode array. The localization experiment (see Sec. 8 in the Supplementary Material) was conducted to demonstrate the mechanical advancements of the localization cap. All analysis presented in the results section use data collected during the hemispheric laterality experiment sessions, unless specified otherwise.

### 2.10 Signal Preprocessing

Minimal analog filtering is applied to the sensor measurements before digitization. The digitized measurement values (6.1 Hz sample rate) are proportional to the incident power detected by the SiPM. This is the starting point for both offline and real-time signal processing. The following digital signal preprocessing steps are used (refer to Sec. 4 in the Supplementary Material for more details):

1. Bad channel removal
2. Detrend
3. Optical density conversion
4. Bandpass filtering
5. Superficial regression
6. Conversion to hemoglobin

### 2.11 Optode Coupling/Pulse Signal-to-Noise Ratio

To quantify the quality of cap-fitting and how well the optodes were coupled to the scalp, we derived individual channel’s pulse SNR from the power spectral density on optical density data, using a constant false alarm rate (CFAR)-based method (see Sec. 5.1 in the Supplementary Material). The pulse SNR measures the strength of heartbeat-related signal within a channel and can be an indicator for optode coupling quality.^36^ The pulse SNR is calculated for all channels that CFAR determines to have a peak in the pulse range (and set to 0 otherwise). Pulse SNR<1 is also set to 0.

### 2.12 Cap Fit Metric

After the subjects donned the custom caps, they shifted the cap and hair around while tightening the sensor modules to achieve a more comfortable fit and better optode coupling to the scalp. We characterized the quality of this cap-fitting process with a custom “cap fit” metric, derived from pulse-SNRs. Pulse SNRs were calculated for all channels with SD-distance between 25 and 50 mm. Channels in this range were chosen as they are most sensitive to cortical activities for all subjects. As there can exist scalp contact differences between individual sensor modules, the pulse-SNR averages for the modules were calculated, and their minimum is the cap fit metric. The rationale for this metric is that better cap-fitting leads to better optode-scalp coupling and stronger pulse detected in more channels.

### 2.13 Trial-Rejection Based on Behavioral Response

For each session, finger tap events represented by keyboard press by the participant were logged. A finger-tapping trial was classified as behaviorally correct only if (1) the correct finger taps (e.g., left fingers for left-hand condition) happened within the expected performance window (during visual cue presentation) and (2) no finger-tap events happened before the onset or 0.8 s after the offset of the visual cue. Behaviorally incorrect trials were discarded from offline analysis.

### 2.14 Characterizing Functional Activation of a Single Channel

To validate that our device was indeed measuring hemodynamic activities, we checked for the resemblance of the two hemoglobin [oxygenated hemoglobin (HbO) and deoxygenated hemoglobin (HbR)] species’ evoked response to the canonical hemodynamic response function (HRF)^37^^–^^39^ and the evoked responses’ consistency with motor hemispheric lateralization.

**Fig. 3:**
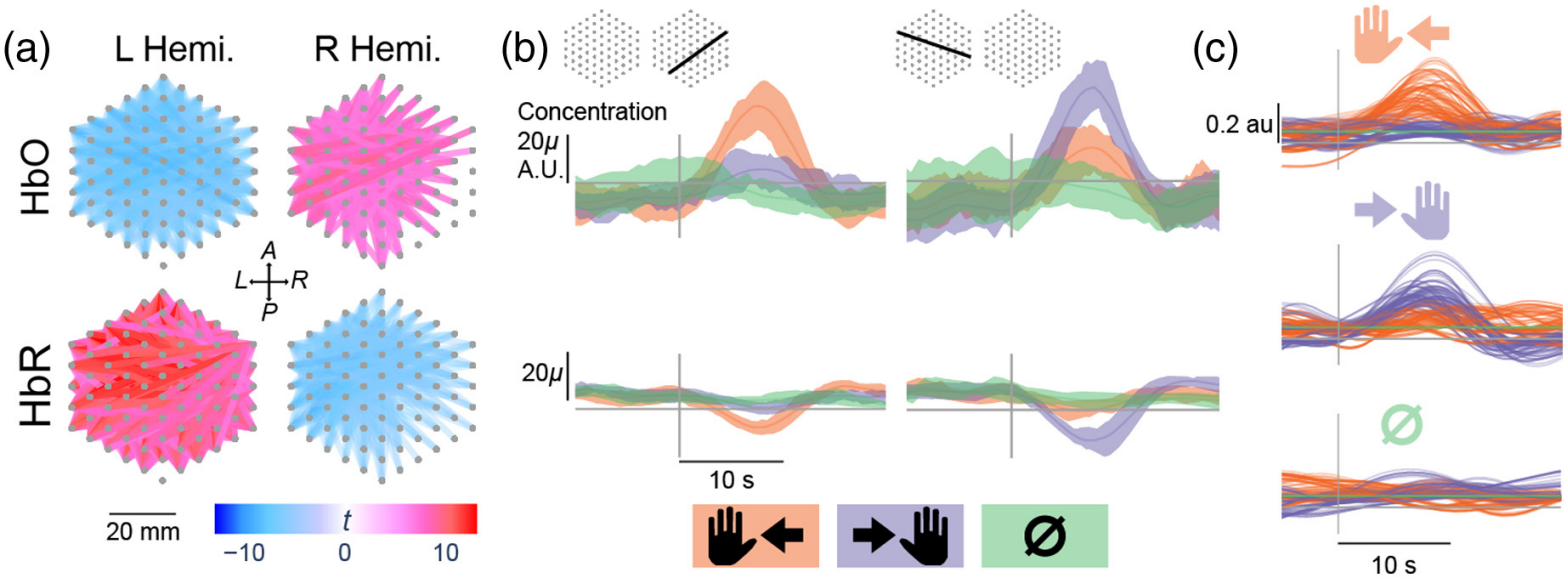
Haystack and evoked response plots: (a) haystack plots showing left-hand versus right-hand tapping contrast t-statistics for modules on the left and right hemisphere for HbO (top) and HbR (bottom). Each hexagonal cluster of points represents the source and detector locations of a single module. Lines connecting two points represent SD pairs, and the line color represents t-statistic values. For HbO, higher t-statistic (more red) indicates a channel is more responsive to left-hand tapping than right-hand tapping. This is the opposite for HbR (more blue is more responsive to left-hand tapping). (b) Evoked response plots showing all finger-tapping conditions for two example channels (left and right columns), with channel locations shown in the module diagrams in the first row. The colored traces and shadings represent the trial average and interquartile range for a channel’s measured concentrations in arbitrary units, different tapping conditions (different colors), and hemoglobin species (different rows). The gray vertical line on the x axis marks the stimulus onset. (c) Decoder predicted hemodynamic response (colored traces) for different trial conditions (top to bottom). Colored traces illustrate the predicted hemodynamic response for different conditions for specific trials (i.e., orange traces in the top plot are the predicted hemodynamic response for hand-right condition when the trial condition is hand-right, lavender traces in the top plot is that for hand-left condition when the trial condition is hand-right).

We used general linear models (GLMs) (see Sec. 5.2 in the Supplementary Material) to measure how much of a channel’s evoked response can be attributed to the canonical HRF evoked by a specific condition (finger-tapping conditions), as a proxy for task-dependent activations exhibited by that channel. The task-dependent activations are quantified by the resulting GLM t-statistics associated with each finger-tapping condition. In addition, one can measure a channel’s task-dependent activation differences with the t-statistic of between condition contrasts. The significant channels and the associated t-statistic values per condition or contrast can then be visualized on haystack plots [see Sec. 2.18 and Fig. 3(a)].

The r-squared ($R^{2}$) of each channel’s fitted regression model evaluates the percentage of variance explained by the expected hemodynamic response for all the conditions and thus can be used to characterize how strong functional responses are for each channel.

### 2.15 Arch Metric: Composite Metric to Measure Captured Hemodynamic Content

**Fig. 4:**
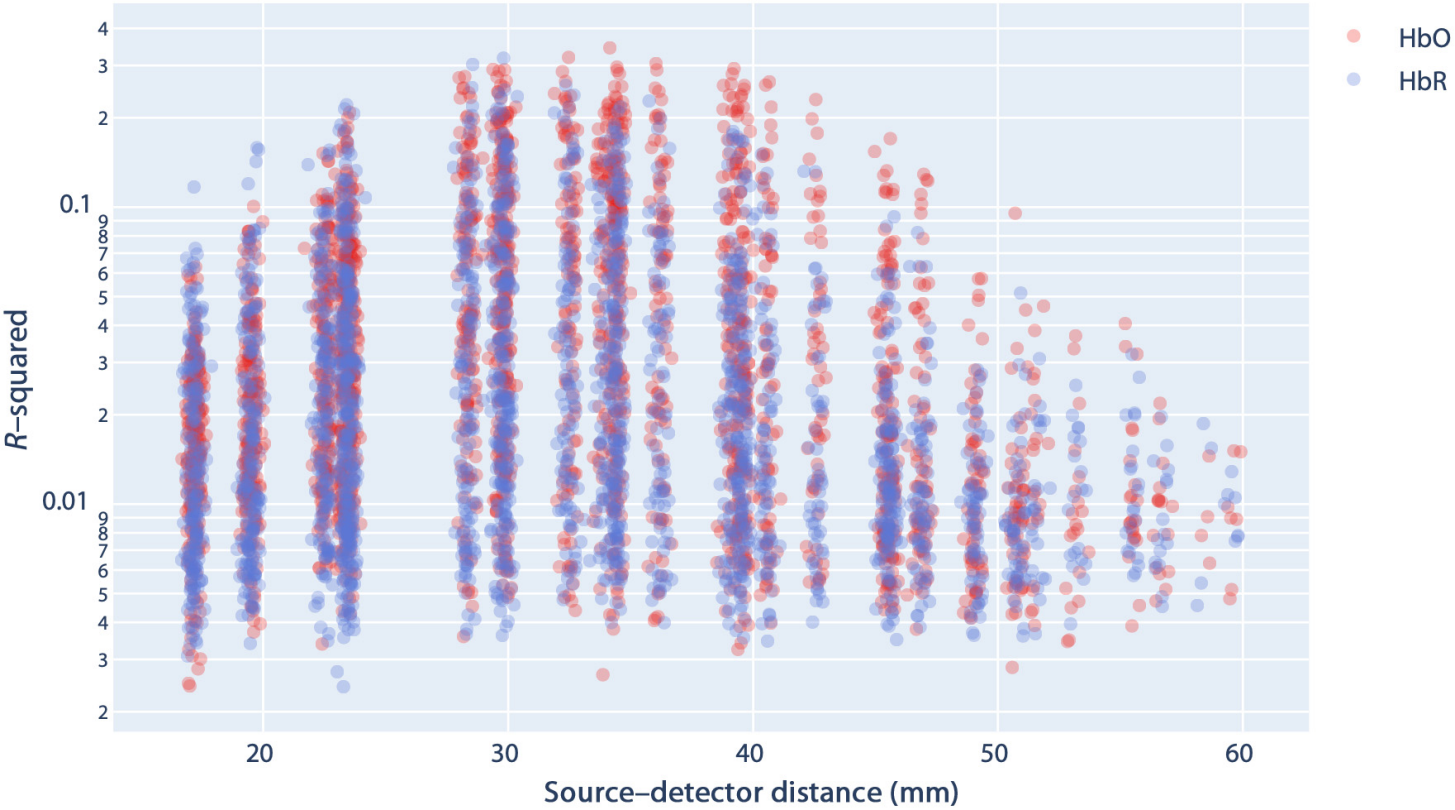
Typical distance versus GLM $R^{2}$ relationship for individual channels. x axis is the SD-distance, y axis is the $R^{2}$ values derived from the GLM regressions. Only values for significant $R^{2}$ values are shown.

A custom “arch metric” was used to quantify “hemodynamic content captured” during an experiment. We assumed that the $R^{2}$ of the regression between a channel’s measurements against canonical HRF can be taken to characterize that channel’s functional activation, if significant. The distribution of the $R^{2}$ values was heavily skewed to the right. The degree of skewness differs for channels within different distance ranges, and the maximum $R^{2}$ values are typically the greatest in distance ranging 25 to 40 mm, before declining toward longer distances (Fig. 4).

To generate a metric for the “amount of hemodynamic content” captured in a recording, we calculate the following “arch metric:”

$$V_{\mathrm{arch}}=\overset{d=55\;\mathrm{mm}}{\underset{d=20\;\mathrm{mm}}{\sum }}\;\max(m(d_{1},d_{2}+5)-m(15,20),0),$$ (1)

where $m(d_{1},d_{2})$ represents the mean of the significant $R^{2}$ values for channels within distance range $d_{1}$ and $d_{2}$. This composite metric provides a more fine-grained way to quantify overall hemodynamic content captured by the system compared to simpler metrics without taking into account channel SD-distance (see Sec. 5.3 in the Supplementary Material). As channel SD-distance increases above 20 mm, the measured signals should become more sensitive to cortical activities. Setting $m(d_{1},d_{2})$ as baseline and summing additional increases in the subsequent SD-distance ranges reflect this property.

### 2.16 Task Condition Decoding

We performed decoding of the finger-tapping experiments’ stimulus conditions both offline from collected data and in real time to provide visual feedback. Pilot experiments have shown motion artifacts present in some channels can be used to decode stimulus conditions and contribute disproportionately to a decoder. To limit the influence of these non-neural related processes, we introduced explicit decoder constraints such that only channels exhibiting HRF-like evoked-responses can be used.

During decoder training, for each stimulus condition, channels were ranked according to their evoked-responses’ resemblance to the canonical HRF. Then for each condition, a linear combination of the top-ranking channels is learned to maximize its correlation with the canonical HRF (termed “predicted hemodynamic response”). During inference, the learned linear combinations are applied and the predicted condition is the one with predicted hemodynamic response most similar to the canonical HRF. Altogether, these constraints maximize the decoder’s reliance on hemodynamic signals (see Sec. 6 in the Supplementary Material).

For offline single-session decoding, we used the first half of the trials as training data and the remaining half as testing data. For real-time, closed-loop decoding sessions, a decoder was trained with two blocks of trials without feedback; then the trained decoder was applied to provide real-time feedback for subsequent testing trials. During decoding performance comparison, a permutation test (n=200) was conducted to approximate a chance decoding level.

### 2.17 Visualization: “Haystack” Plots

Given a scalar statistic (e.g., the t-statistics from GLM) for each SD pair, we can visualize them spatially in a “haystack plot” [Fig. 3(a)]. Each SD pair is represented by a line connecting the coordinates of the corresponding source and detector locations in a 2D plane, with the line color representing the scalar value.

In Fig. 3(a), the two modules are plotted in the same coordinate frame, roughly as if we flattened the curved surface of the skull in which the modules were mounted. The origin of this coordinate frame is the midpoint between the two modules. The x axis corresponds to the left-to-right direction, and the y axis corresponds to the anterior–posterior direction.

## 3 Results

We first present *in vitro* device characterization followed by cortical activities measurement and decoding for *in vivo* finger-tapping experiments [Fig. 1(d)].

### 3.1 Sensor Characterization

The average optical power measured at the end of source ferrules was 35.2 mW per red LEDs (680 nm) and 30.9 mW per infrared LEDs (850 nm). The average coupling efficiency of the source ferrules, which measures the percent of optical power transmitted from the LED, was 23.4% for red wavelength (680 nm) and 26.8% for the IR wavelength (850 nm). The average coupling efficiency of the detector ferrule, which measures the percentage of optical power transmitted to the detector via the ferrule, was 11%. Detector SiPM NEP was calculated from datasheet values to be 1.27 fW-rms (850 nm) and 0.77 fW-rms (680 nm), translating to detectivity^34^ of 40.4 and $24.5\;\mathrm{fW}\text{-}\mathrm{rms}/{\mathrm{mm}}^{2}$ (see Sec. 1.3 in the Supplementary Material).

### 3.2 System Performance Benchmark

The metrics (see Sec. 2) presented here are the median of all measured sensor modules calculated from phantom experiment data. The module median detector noise density is 17.7  fW/rtHz and is calculated from optode pairs with SD-distance >55  mm. This noise figure is low compared to conventional CW-fNIRS systems using photodiodes or APD. The module median detector power is 51.45 pW and is calculated from optode pairs with SD-distance within [27, 33] mm. We characterized optode pairs to be “good” when they have SNR>100 and tracked the percentage of good channels within the [27, 33] mm SD-distance range, which was over 99.0% for all modules tested (results in ∼505 channels out of 510 in the range). As further comparison, we also calculated the number of viable channels using definitions presented in Refs. 40–42. We calculated the noise floor from channels with SD distances above 55 mm and used that as a threshold on phantom data, yielding 2984 viable channels (∼93.3% of 3198 channels). The overall system dynamic range was measured to be 120 dB.

### 3.3 Task-Dependent Hemodynamic Responses Consistent with Motor Cortical Hemispheric Lateralization

GLM analysis to characterize functional activation was applied on the hemispheric laterality experiment data. Figure 3(a) plots the t-statistics of left-hand versus right-hand tapping condition contrast for different channels spatially, in the form of “haystack plots,” for each module (left versus right column) and hemoglobin species (top: HbO and bottom: HbR). For clarity, within a module, only channels with t-statistic in the top 50-percentile were shown, and each channel’s t-statistic value is represented by the color of the line connecting that channel’s source and detector locations. The plotted values indicate how much the concentration of the hemoglobin species for each channel can be explained by the expected hemodynamic response due to left-hand tapping compared with that due to right-hand tapping. The concentration of HbO is expected to increase during cortical activations and correlate positively with canonical HRF and vice versa for HbR.^37^^,^^43^ Motor cortical hemispheric lateralization predicts that the left motor cortex should exhibit stronger activation during right-hand tapping and vice versa. This means that the t-statistic for left-versus-right contrast should be negative for left-hemisphere HbO channels (less left-hemisphere activation during left tapping) and positive for right-hemisphere HbO channels (more right-hemisphere activation during left tapping). This trend should be reversed for HbR channels. Indeed, this is the case as shown in the haystack plots.

The evoked response plots in Fig. 3(b) illustrate how HbO and HbR concentration time courses differ for different tapping conditions, for a representative channel from each hemisphere. The HbO time courses for both channels correlate with that of the canonical HRF, but the peak for left tapping (orange trace) is greater than that for right tapping (lavender trace) in the right hemisphere channel (top-left plot) and vice versa for the left hemisphere channel (lavender peak higher than orange peak in top-right plot). The trends for HbR (bottom plots) are reversed for each channel, as expected of the anticorrelation between HbO and HbR.^43^

One motivation of having higher optode density in an fNIRS system is the potential ability to resolve finer spatial differences in hemodynamic activities. We conducted a variant of the hand tapping experiment to check if Spotlight can differentiate functional activations corresponding to fingers on the same hand without using tomographic reconstruction (see Sec. 7 in the Supplementary Material). Despite some marginal trend in the HbO activation in differentiating tapping right thumb and pinky fingers, we did not observe significant separation and found no clear positive evidence.

### 3.4 Task Condition can be Decoded from Hemodynamic Responses

Figure 3(c) shows the different predicted hemodynamic responses for different stimulus conditions in one offline decoding session. This figure illustrates that the predicted hemodynamic responses for the true stimulus condition are usually the ones that most resemble the canonical HRF and that the decoder is most likely leveraging hemodynamic activities.

**Fig. 5:**
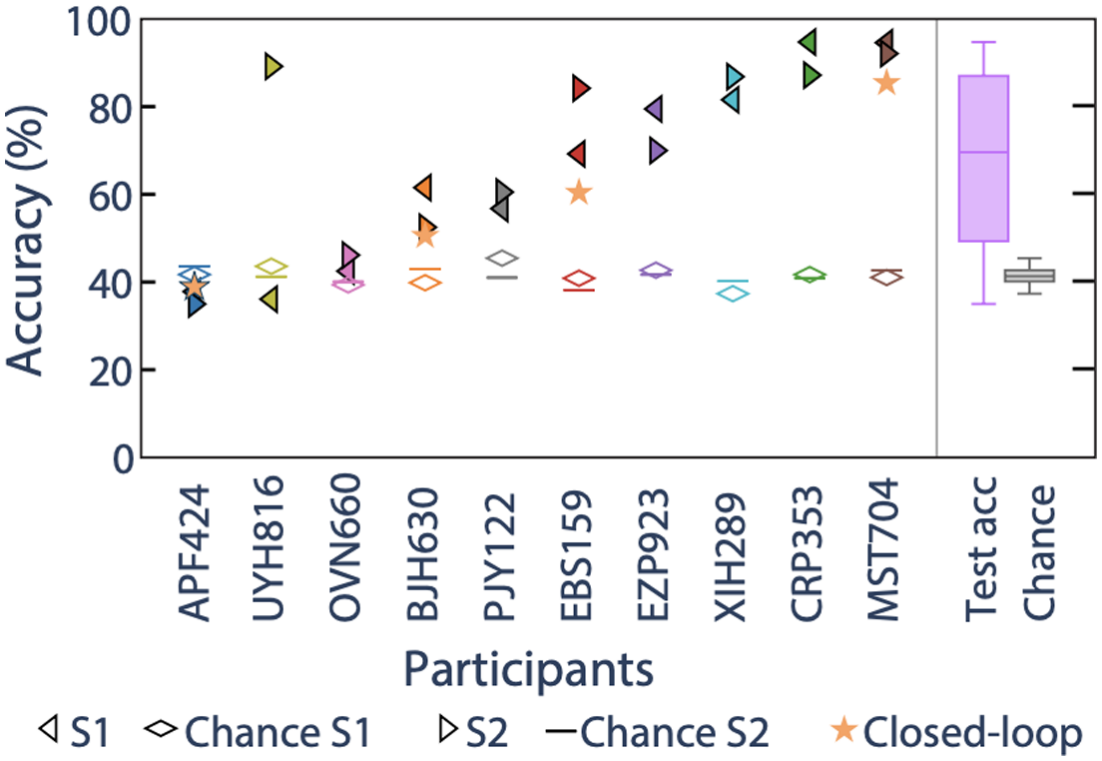
Finger tapping decoding accuracy across subjects for two sessions each (S1, S2, filled triangles). Diamond and horizontal lines correspond to the permutation test decoding accuracies (chance level) for the same sessions. Subjects are sorted on the x axis by their lowest session decoding accuracy (low to high). The y axis is decoding accuracy in percentage. Four subjects (subject APF424, BJH630, EBS159, and MST704) also each performed a session where real-time decoder feedback was given, and the real-time decoder results are shown as stars. The lavender boxplot aggregates all offline decoding accuracies across subjects and non-feedback sessions (25th percentile = 49.3%, median = 69.6%, 75th percentile = 87.0%). The gray boxplot aggregates that for permutation test decoding accuracies.

Figure 5 shows the offline decoding accuracy for all participants in the Hemispheric Laterality Protocol. The median decoding accuracy across 10 subjects was 69.6%, ranging from a minimum of 35.0% to a maximum of 94.7%. The chance decoding accuracy, calculated as the median permutation test decoding performance across all the subjects and sessions, was 41.3%, and 15 out of 20 total sessions had decoding accuracy significantly above their corresponding chance level. Between-session decoding performances differences were small (<10%) for all but 2 subjects (UYH816 and EBS159).

Four subjects additionally performed the Hemispheric Laterality Protocol with real-time feedback provided by a decoder trained from two experiment blocks. The real-time decoding accuracies are similar to each subject’s respective offline decoding accuracies (see Fig. 5).

### 3.5 Better Cap Fit Correlates with More Captured Hemodynamic Activities and Higher Decoding Accuracy

The simplicity of our finger-tapping validation task and the decoder structure allowed us to analyze factors that affect Spotlight’s system performance in the BCI context. We hypothesized more hemodynamic content captured should lead to better decoding accuracy and factors, such as cap fit and personalization would contribute to more hemodynamic content to be captured.

**Fig. 6:**
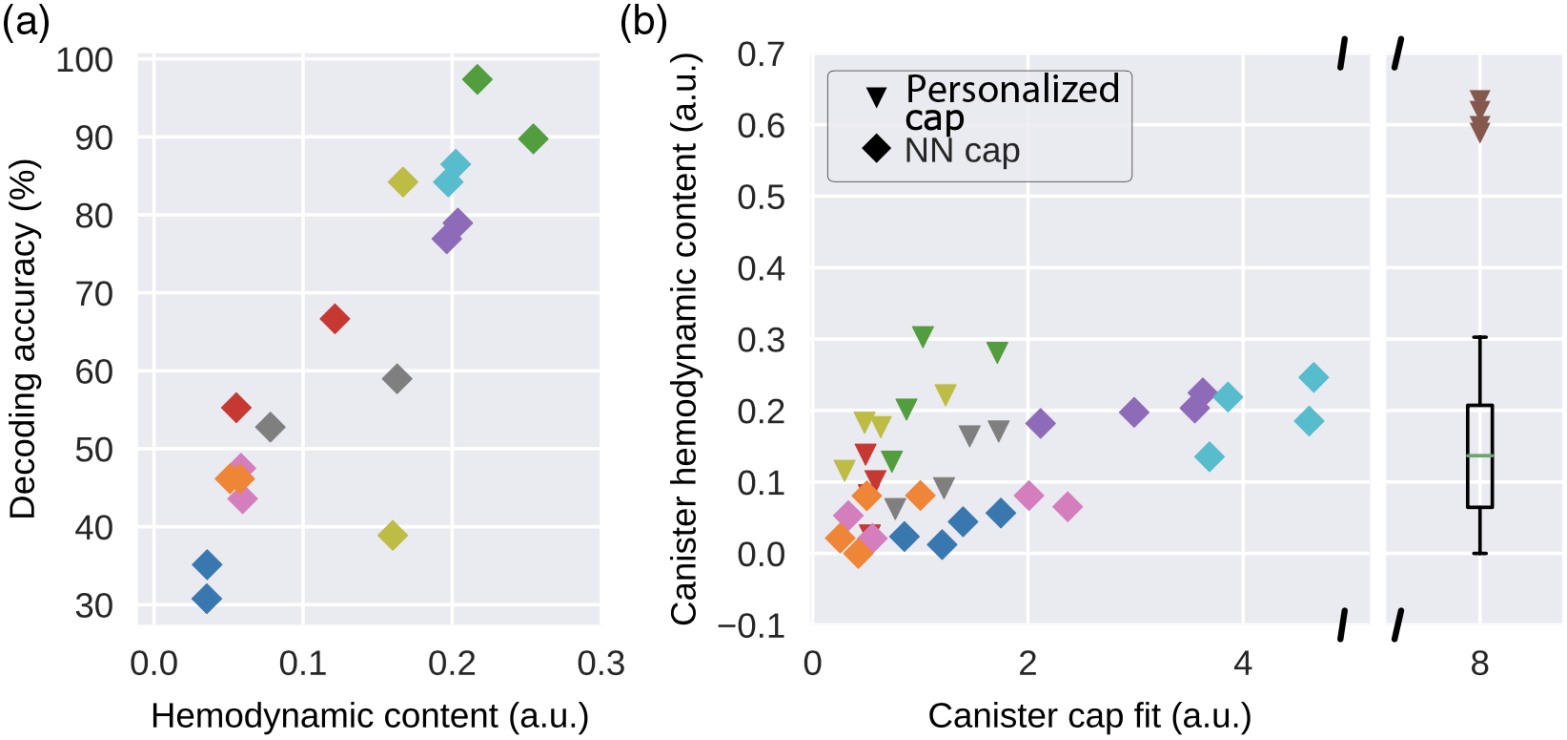
Better cap fit correlates with more captured hemodynamic content and higher decoding accuracy: (a) hemodynamic content captured by all sensors correlates strongly with decoding accuracy ($R^{2}=0.761$, $p<5\times 10^{-6}$). Each point represents a single session, colors represent different subjects, and the color corresponds with Fig. 4. (b) Scatterplot shows cap fit versus hemodynamic content captured for individual modules. Each point represents a module in a single session, colors represent different subjects, and shapes represent whether a personalized cap was used (NN, nearest neighbor cap). Regression between cap fit and hemodynamic content has $R^{2}$ of 0.249 (p<0.001), which increased to 0.590 ($p<5\times 10^{-7}$) after taking into account of the cap type. The boxplot shows the distribution of module hemodynamic content, with the brown triangles representing outlier sessions for a single subject that were excluded in the regression analysis.

We treated the $R^{2}$ of each channel’s HRF GLM (see Sec. 2) fit as a measure of its functional activation. A custom “arch metric” was used to quantify “hemodynamic content captured” during an experiment (see Sec. 2.15), which summarizes the functional activation of channels over different SD-distance ranges. As this quantity is heavily skewed to the right, we further apply a square root transform in regression analysis and this transformed quantity is referenced below and in Fig. 6.

We found that the total hemodynamic content captured by all the channels correlated strongly to offline finger-tapping decoding accuracy [$R^{2}=0.761$, $p<5\times 10^{-6}$, Fig. 6(a)]. As the two modules on a single cap can be adjusted with some degrees of freedom, we found that individual module’s cap fit (see Sec. 2.12) correlated moderately with the corresponding module’s hemodynamic content captured ($R^{2}=0.249$, p<0.001). However, the per module relationship between cap fit and captured hemodynamic content increased significantly after taking into account different subjects’ cap personalization [$R^{2}=0.590$, $p<5\times 10^{-7}$, Fig. 6(b)]. Specifically, given the same cap fit metric, the usage of a personalized cap correlated with an increase in captured hemodynamic content.

Since cap fit measures scalp-optode coupling and captured hemodynamic content measures task-dependent activation, the positive effect of cap personalization shown was interpreted to indicate that personalized caps enable better placement of the modules over the hand-motor cortical areas. Subsequently, we tested the effects of non-centered placement of the module over the hand-motor cortex for one subject with a personalized cap based on functional MRI data in a cap localization experiment (see Sec. 8 in the Supplementary Material). This experiment showed significant differences in task-dependent activations among different module placements, with centered placement resulting in higher activations for more channels (Fig. S27 in the Supplementary Material), consistent with our interpretation of the cap personalization effect.

As an additional control, we also performed regression analysis including hair length as a factor (six subjects with short hair, three subjects with medium hair, and one outlier subject with shaved head). Hair length was found not to be significantly correlated with decoding accuracy, cap fit, or captured hemodynamic content. It was also found not to be significantly correlated with hemodynamic content after taking into account cap fit and/or cap personalization.

## 4 Discussion

The Spotlight system presented here advances upon the current portable high-channel count fNIRS systems by increasing the optode density, lowering noise floor, and maintaining system SNR and dynamic range, such that it approaches fibered high-density systems’ performance while staying portable. Each of Spotlight’s 60 mm-diameter modules fits 80 optodes with 6.5 mm inter-optode spacing. Commercially available portable CW-fNIRS systems have larger inter-optode spacing and a sparser set of total available channel SD-distances. For example, Lumo provides channel SD-distances of 10, 20, 50, 60 mm, and above; NIRX CW-fNIRS provides channel SD-distances of 15, 34, 54, and 64 mm. In comparison, our system has more than 20 different channel SD-distances between 6.5 and 60 mm, with ∼60% of channels in the SD-distance range 20 to 40 mm. Previous studies using different Lumo configuration in 12-module and 24-module systems quantified the number of viable channels available and found 489 channels (dual wavelength) out of 1728 channels *in vivo*,^40^ 717 per wavelength out of 1728 channels (in phantom),^41^ and 800 channels out of 3456 dual wavelengths channels (in 10 to 45 mm).^42^ Although it is difficult to directly compare systems with different optode geometry and configuration using this metric, Spotlight had an average of 2984 out of 3198 channels (∼93.3%) that were viable in phantom measurements.

Spotlight’s improved sensor packing and performance can largely be attributed to SiPM’s single photon sensitivity, fast timing response, low operation bias, and compactness,^44^^,^^45^ combined with integrated optomechanical ferrule design. Spotlight adds to recent works in multichannel fNIRS systems based on FPC-integrated SiPMs^46^^–^^49^ that altogether point to SiPM detectors as a clear direction for future works.

The optical improvements enabled us to miniaturize high-channel count sensor assemblies into a small, portable modular form factor that simplifies fNIRS-based neuroimaging and BCI experiment setups. Although we have only used two modules at a time in the presented experiments, multiple modules can combine to easily increase imaging coverage area when combined with 3D cap printing.

Although our human subject experiments demonstrated that a single-user operated Spotlight can reliably capture task-dependent hemodynamic responses that can also be used for real-time decoding, they also provided insights into areas of improvements needed for fNIRS technology, particularly toward BCI applications. Perhaps unsurprisingly, we found that better optode-coupling, as measured by our cap fit metric, is associated with more captured task-dependent hemodynamic response, leading to better decoding accuracy. The lack of cap fit seems to be mitigated using personalized caps that can better center the sensor modules over the cortical areas of interest. The effect of localization can be quite significant as demonstrated by our localization experiment and points toward potential experiment protocols where the modules’ placement can be adjusted in real time. Neither cap fit, task-dependent activation, nor decoding accuracy seem to be significantly associated with hair length, which suggests our flexible-membrane embedded optode design may be effective at combing through hair and making effective scalp contact. Although further studies with more diverse hair characteristics are needed, this points to membrane-structure as an alternative to NIRx-style individually spring-loaded optode design for effective light-coupling to the scalp. Adjustable module placements and conforming membrane optode structures combined with more adjustable cap designs should then make it possible to conduct very large-scale fNIRS studies with diverse populations, while maintaining overall signal quality, portability, and ease of use.

One of Spotlight’s key strengths is its high optode density and channel counts. Studies with the current state-of-art fiber-based HD-DOT systems have shown improvements in spatial resolution,^22^ SNR,^23^ depth sensitivity, and specificity.^24^ Of these potential improvements, better spatial resolution is of particular interest to improve the ability of fNIRS to study cortical processes and their application to BCI. Using ultra high density DOT, Markow et al.^50^ discussed how the point-spread function decreased as the optode density increased, demonstrated that a 6.5 mm-spaced optode DOT system covering visual cortex yielded higher decoding accuracy than their previous 13 mm-spaced DOT system in decoding naturalistic movie clips seen by human subjects, and attributed the decoding improvements to better spatial resolution associated with the higher optode density. A key limitation of BOLD imaging techniques, such as fNIRS and fMRI, has been their low temporal resolution, restricted by the relatively long time course of the hemodynamic response. Although ongoing research aims to improve the temporal resolution by potentially leveraging the “initial dip” feature^51^ of the hemodynamic response, a promising alternative approach is to derive the time course of cortical responses by tracking the phase differences of hemodynamic response across cortical locations,^52^ effectively achieving higher temporal resolution. Therefore, increasing the optode density of an fNIRS system may lead to increase in both spatial and temporal resolutions.

Our supplementary experiment checked Spotlight’s ability to differentiate activation patterns corresponding to the tapping of different fingers on the same hand, hypothesizing its optode density should provide enough spatial resolution for this task. The lack of clear positive evidence in this supplementary experiment does not reject the value of having higher optode density, however. Higher optode density relaxes the sensor localization requirements and allows the real-time localization concept mentioned before to be potentially applied on the module level by operating only the most task-activated channels with longer integration windows to increase SNR. Spotlight’s inability to differentiate same-hand finger-tapping responses also point to future lines of investigation for increasing fNIRS spatiotemporal resolution. We did not apply full tomographic reconstruction as in Ref. 50. Somatotopy and functional organizations in the motor cortex, especially in the hand-motor cortex, are spatially convoluted and functional areas contain significant overlaps,^53^^–^^56^ such that sensor-space analysis and naive infinite-slab tomography models may not be sufficient to distinguish the responses. Additionally, the activation differences for same-hand finger-tapping are likely to be less than different hand tapping, thus requiring significantly more trials to be collected. Taken together, future validation of fNIRS spatiotemporal resolution improvements due to optode density should consider more carefully choices in cortical regions of interest and signal analysis spaces.

Spotlight’s goal is to provide a more portable, accessible, and powerful fNIRS device for neuroscience and BCI applications. As we share Spotlight designs, we hope it can spur more advances in fNIRS technology and better enable future non-invasive neuroscience and BCI research.
