# Supplementary material for: Scalable, modular continuous wave functional near-infrared spectroscopy system (Spotlight)
Source: J Biomed Opt. 2023 Jun 13;28(6):065003. doi: 10.1117/1.JBO.28.6.065003 (PMC10261976; doi:10.1117/1.JBO.28.6.065003)
Supplement: Supplementary file 1 [file JBO_028_065003_SD001.pdf]

Supplementary Materials for

Scalable modular continuous wave functional near-infrared spectroscopy system (Spotlight)

Daniel Anaya[1†], Gautam Batra[1†], Peter Bracewell[1†], Ryan Catoen[1†], Dev Chakraborty[1†], Mark Chevillet[1†], Pradeep Damodara[1†], Alvin Dominguez[1†], Laurence Emms[1†], Zifan Jiang[1†], Ealgoo Kim[1†], Keith Klumb[1†], Frances Lau[1†], Rosemary Le[1†], Jamie Li[1†], Brett Mateo[1†], Laura Matloff[1†], Asha Mehta[1†], Emily M. Mugler[1*†], Akansh Murthy[1†], Sho Nakagome[1†], Ryan Orendorff[1†], E-Fann Saung[1†], Roland Schwarz[1†], Ruben Sethi[1†], Rudy Sevile[1†], Ajay Srivastava[1†], John Sundberg[1†], Ying Yang[1†], Allen Yin[1†]

*Corresponding author. Email: mugler@meta.com

This PDF file includes:

Supplementary Text
Figs. S1 to S29
References

We provide design details of our modules with the necessary information for reproducing our work. Mechanical, optical and electrical subsystem assemblies are described in depth, followed by software and firmware architectures. We include additional detail in the signal processing pipeline, and additional experiments beyond what was covered in the main paper, including the experiment setup and results of single digit experiment and localization experiments.

1. Opto-mechanical design

The content of each Spotlight module is shown in Figure. 1A. Figure S1 shows actual shots of the finished assembly, exposing the Flexible Printed Circuits (FPC), analog-to-digital-converter ADC/control boards, optical ferrules and the flexible membrane. Figure S2 shows possible multi-module arrangements.

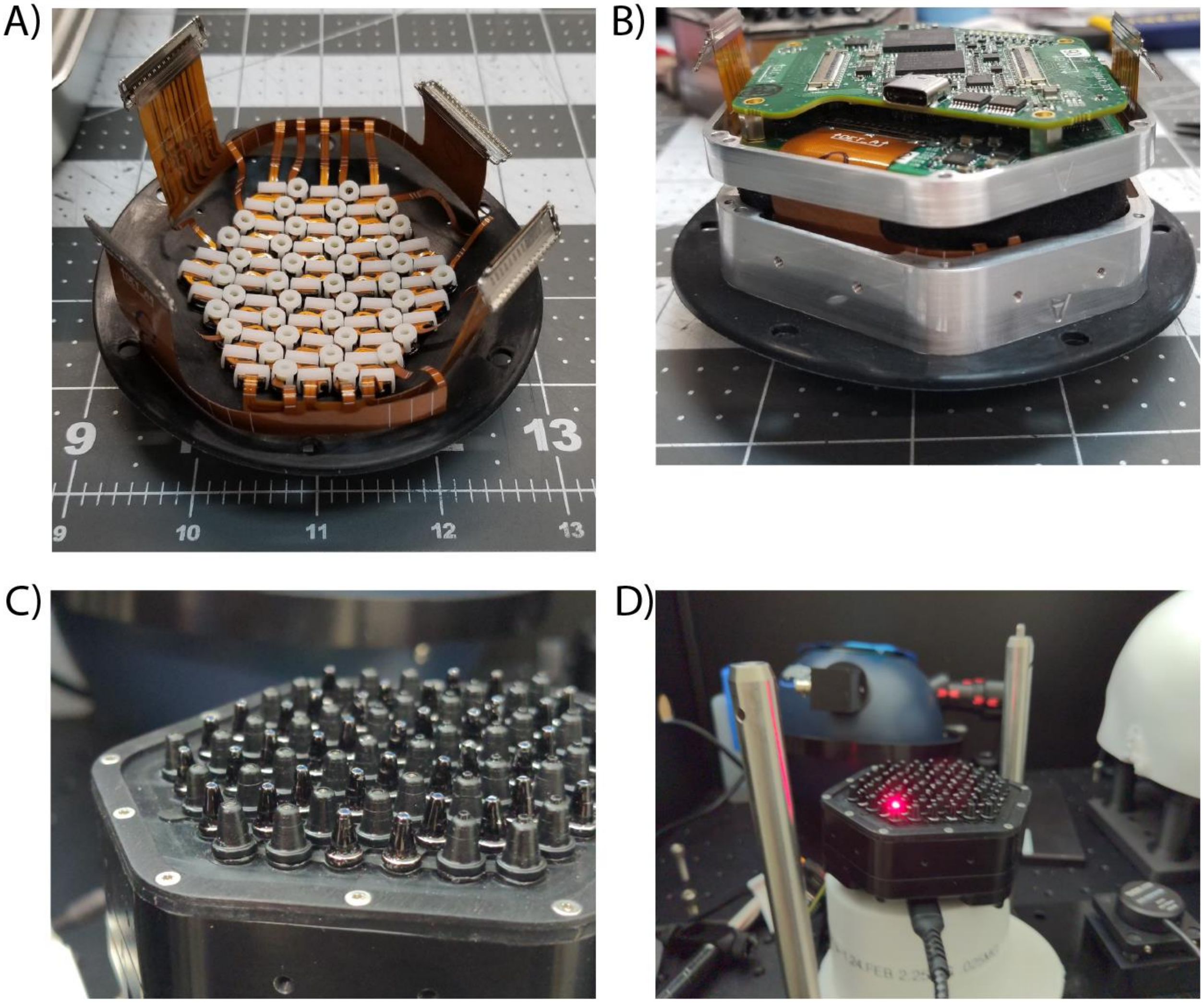

Figure S1. A) Detector and source FPCs folded in the enclosure on top of the membrane. B) Mainboard and ADC board assembled on top of the FPCs. C) Close-up of the optical ferrules embedded in the flexible membrane. D) Assembled module in black ABC housing. The gap at the bottom allows for flush USB connection.

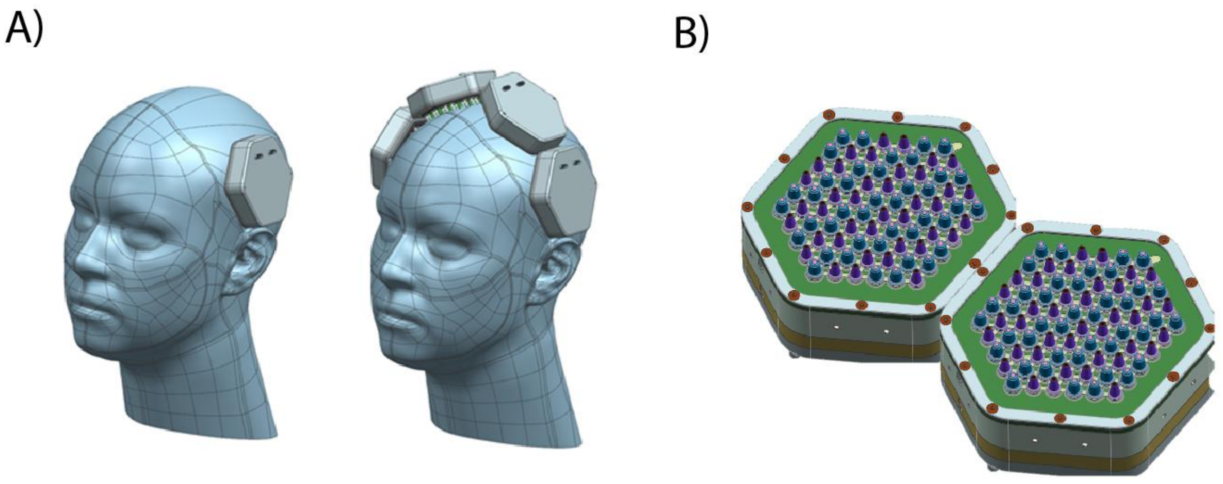

Figure S2. One or more Spotlight modules can be placed over the head. A) Example CAD rendering of placements of modules along the sensorimotor cortices. B) The close-up shows the smallest inter-module gap of 25 mm due to module housing thickness.

1.1 Optical ferrules

The ferrules are the core mechanical interface between the source/detector and the head. The design involved balancing the following priorities: 1) Prevent losses through increased coupling efficiency 2) The outer form of the ferrule to "comb" through the hair 3) Reduce overall ferrule size to increase source detector efficiency 4) Use materials that are biocompatible and effectively block stray light when needed.

The ferrule housings needed to be minimized to maximize packing and provide a structural yet flexible support for the membrane. This resulted in the ferrule tips being of a conical shape while still maintaining 2 mm contact diameter at the skin. The pitch distance between each source and detector is 6.5 mm as in Figure S6.

1.2 Detector ferrule

The assembly of the detector ferrule is shown in Figure 1 B (main text) and also in more details in Figure S3. The SiPM detector (OnSemi RD10035) has an active detector area of 1 mm x 1 mm square. A 800 um diameter silica core fiber diced at 10.75 mm fits inside the detector ferrule housing. The housing is made of a polycarbonate/ABS mixed material. The detector is surface mounted onto the FPC. A dual band optical filter (685DIB1515-C, Knight Optical U.S.A), which passes through at 640 – 730 nm and 810 – 910 nm was diced to 1.5 mm squares, and adhered to the detector using a low viscosity, UV-based adhesive, Norland Adhesive NOA86H. The capillary action and surface tensions around the edges of the detector centered the fiber during the UV cure. The ferrule is assembled separately with a 800 um silica core with UV adhesive and attached using the clip to retain the part onto the FPC. This fiber diameter was chosen as to not overfill the detector with the offset distance equal to the thickness of the optical filter.

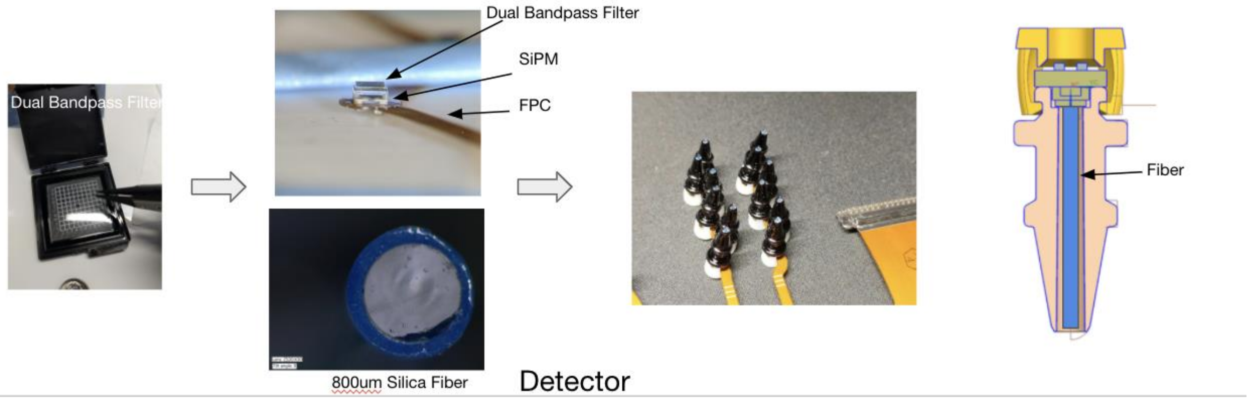

Figure S3. Detector ferrule assembly process. The dual bandpass filter diced into 1.5 mm squares is attached to the SiPM via adhesive. SiPM sensors are surface mounted onto the FPC. The 800 um fused-silica fiber is bonded to the black polycarbonate ferrule housing via EFD. The ferrule housings are then bonded to the FPC-sensor stacks with EFD. The entire ferrule assemblies are finally secured with polycarbonate/nylon clamps (yellow in the schematic at right).

1.3 SiPM Noise-equivalent Power density (NEP) calculation

Typically SiPM device datasheets do not provide noise value in the form of NEP because they are not equivalently calculated as APD or photodiodes. The datasheet for the device provides dark count rate but not the Photon Detection Efficiency (PDE) and responsivity values, so the PDE of the OnSemi Micro RB10035, which is representative of OnSemi Micro RD10035 was used. Here we show how to estimate the SiPM's noise floor based on dark count rate and PDE.

The relationship between photon count rate and optical power can be determined by:

1) The energy per photon can be calculated through Planck's equation, which yields 2.337e-19 J/photon (@850nm), and 2.921e-19 J/photon (@680nm).
2) Micro RD10035 has mean dark count rate of 850 kcps.
3) The root-mean-square (RMS) uncertainty of the dark photon rate follows Poisson distribution and is equal to ~922 (square root of 850kcps).
4) The SiPM dark current power (shot noise) is then obtained by multiplying (1) and (3), leading to 0.215fW-rms (850nm) and 0.269fW-rms (680nm).
5) These numbers assume perfect PDE and need to be divided by the actual PDE value (17% for 850nm, 35% for 680nm), yielding 1.27fWrms (850nm) and 0.77fWrms (680nm). These are the SiPM NEP values presented in the main text.

To obtain the detectability metric used in the paper [34], we followed their procedures and divided the NEP values by the cross-section area of a 200um-diameter optical fiber, yielding 40.4fW-rms/mm^2 and 24.5fW-rms/mm^2.

This detector NEP was calculated assuming a single SiPM receiving light continuously directed to the detector surface. During actual Spotlight module operation, 39 detectors are being time multiplexed, thus reducing the sensor integration time.Furthermore, the coupling efficiency of each detector ferrule is about 11%, meaning much of the light hitting the optode does not reach

the SiPM active surface, resulting in a smaller signal level. Combining the reduced integration time, and lower signal level, we would expect the system SNR to reduce by a factor of sqrt(39*2/0.11)=26.7 relative to that of a continuously operating SiPM. and we should expect a system level NEP of 26.7fW/rtHz. As the measured 17.7fW/rtHz is smaller than 26.7 fW/rtHz, it might suggest that the noise performance specified in the datasheet is an underestimate.

1.4 Source ferrule

The goal of the source ferrule and its content is to maximize the coupling of the power output from the LED in the 2 wavelengths of interest (680 nm, 850 nm). Several ferrule coupling designs were evaluated using a non-sequential Zemax model (boundary conditions: source is 0.225 mm off axis, detector is a 1.5 mm-by-1.5 mm square set 1 mm from the source output) with a dimensionally representative dual source LED from Marubeni (part number 680/850D-23), which provided upper-bound coupling efficiencies we can expect. The LED (Figure S4) has a beam profile shown in Figure S4B, with a nominal divergence angle of +/-15 degrees, therefore additional focusing mechanism inside the source ferrule housing was needed to maximize light output. The simulated design with the best coupling efficiency (74 %) was replacing the flat epoxy lens covering the LED with a ruby ball lens to focus the source radiation through a polycarbonate light pipe. However, this required significant supplier repackaging and introduced possibilities of debris from the assembly process. The final source ferrule design is shown in Figure 1B and Figure S5, which uses a custom polycarbonate light-pipe (Sabic HP-1 with diamond point turned tooling) inside the ferrule housing, narrowing toward the ferrule opening, to focus the LED light output. Factors of light losses such as fresnel reflections exist at the interfaces due to change in index of refraction. The mismatch between the LED lens and the light pipe was mitigated by applying a broadband anti-reflective (BBAR) coating that is 99 % anti-reflective for wavelengths between 650-1000 nm at the LED end. The ferrule opening end of the lightpipe was left uncoated since it will be in contact with the skin and needs to be biocompatible.

A)

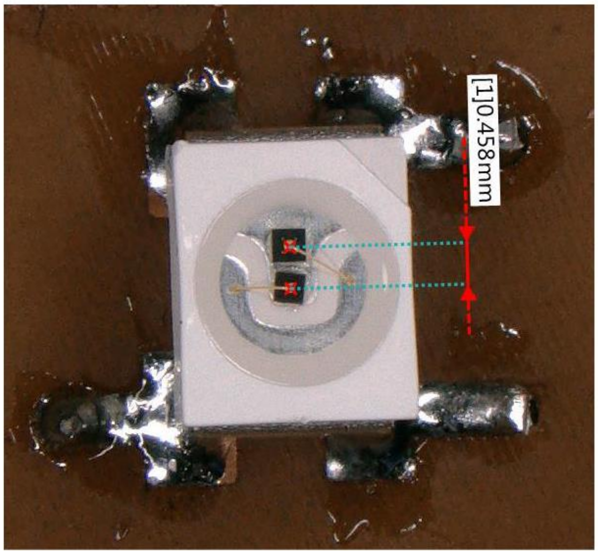

B)

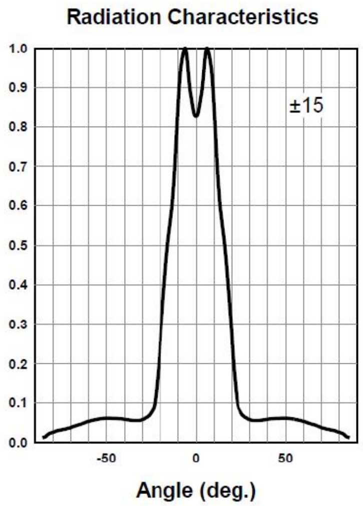

Figure S4. A) Marubeni (680/850D-23) dual source LED physical dimension. The measurement shows the distance between the different sources. B) Beam profile with FWHM at +/-15 degrees.

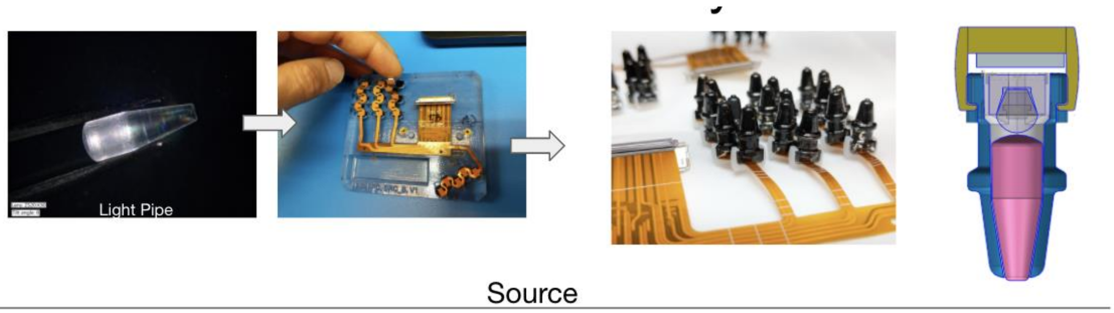

Figure S5. Source ferrule assembly process. The polycarbonate light-pipe (left figure, pink in the right schematic) and the LED (bulb with blue outline in right schematic) are press-fitted into the black polycarbonate ferrule housing. The ferrule housings are then bonded to the FPC with the help of a flat shim (second figure to the right), before being secured with polycarbonate/nylon clamps (yellow in the schematic at right).

1.5 Ferrule housing

The source and detector housing were molded with a polycarbonate/ABS material mixture called PC/ABS EMERGE PC/ABS 7100 MED that is considered biocompatible. Since polycarbonate is transparent in the IR, the polycarbonate/ABS mix has qualitatively worked well.

1.6 Flexible membrane

Optical ferrules are embedded in a silicone membrane which provides flexibility to the optode position/direction for various head curvatures. In order to do this, a head curvature analysis (Figure S6A-B) was done using head models (*67*) from the National Institute for Occupational Safety and Health (NIOSH) to derive membrane parameters (Figure S6C) to accommodate 95 % of the population.

We assumed a single module coverage area of roughly 62 mm diameter considering active cortical cortex area, person to person variation, and uncertainty in localization. From the outer diameter of the module, the total range of deflection was measured. This measured range will correlate to the total required displacement, from center to edge. With a 52 mm diameter probe done in 3D CAD NX, the total displacement of 6.7 mm was measured.

The membrane is made of a compression molded silicone with a Shore 40A hardness with a thickness of 2 mm. The membrane is pre-stretched about 3 % before being clamped to prevent the membrane from entering into a bi-stable state, domed upward or inward. One of the challenges of the membrane is to provide flexibility, yet also be a structural member to retain the ferrule. The pushout force of the ferrule to membrane was measured to be 560-600 g, and proved to be sufficient in preventing ferrule push out with a variety of participants. Since polycarbonate is undesirably transparent in the IR, we chose a polycarbonate/ABS material mixture that is IR opaque for the light-blocking ferrule housings.

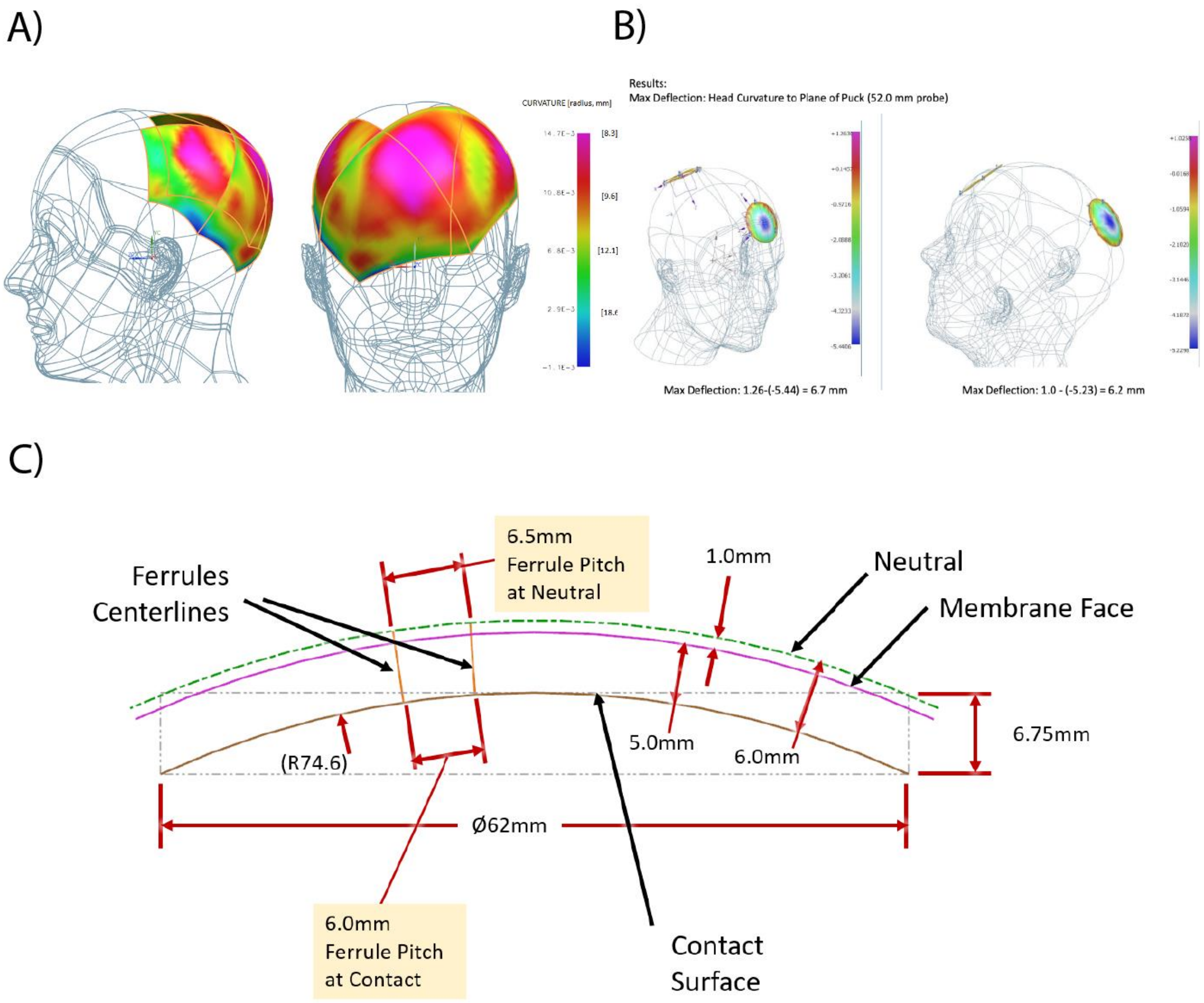

Figure S6. Head curvature study for deriving membrane requirements: A) The range of human head surface curvatures was derived from NIOSH head models. The color represents the magnitude of curvature. B) Example locations of module placements are shown with the curvature at those locations. C) Derivation of the flexible membrane parameters from maximum head curvature and module coverage area (diameter = 62 mm), showing a maximum membrane displacement of 6.75 mm.

2. Electrical design and sensor characterization

2.1 Flexible Printed Circuits (FPCs)

The electrical circuit that interfaces the optical components (SiPMs and LEDs) need to be minimized as much as possible to maximize optode density. In order to provide flexibility to the assembled sensor module of the optodes we designed the FPCs in the shape of a tree-branch structure as in Figure S7. Four FPCs were used in total per module: 2 for the sources and 2 for the detectors. The FPCs are folded and interleaved to fit within the module housing.

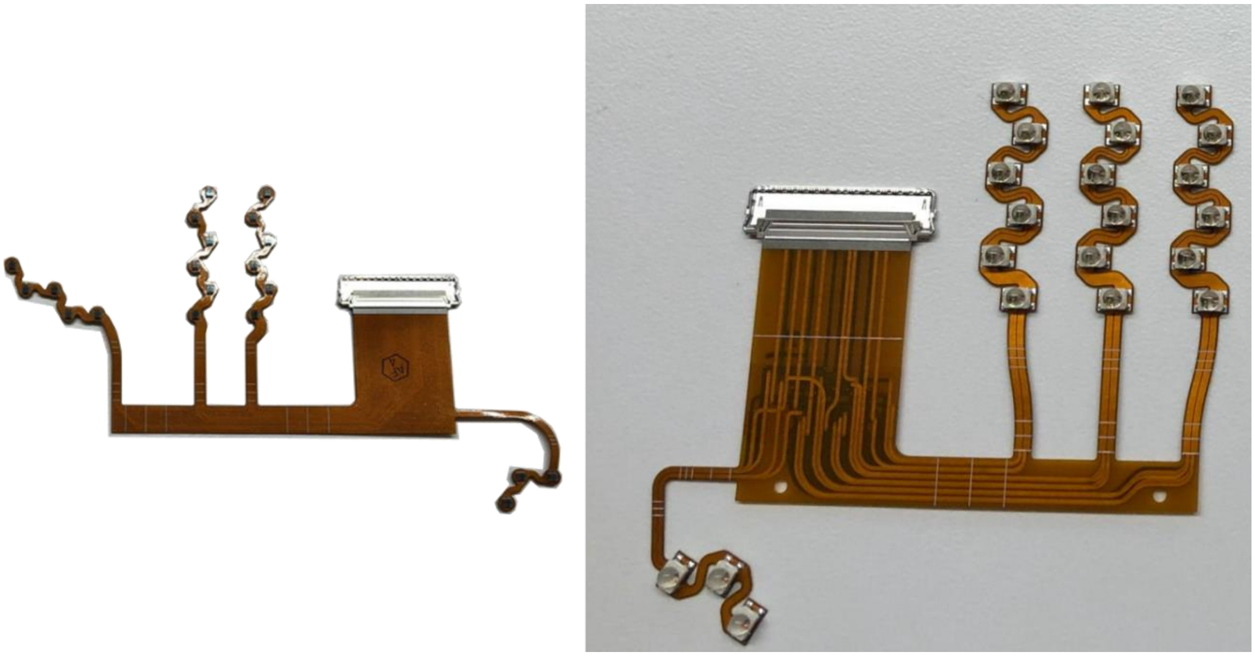

Figure S7. Left: Detector FPC with 20 surface-mounted SiPM on 4 branches. Right: Source PFC with 20 mounted LEDs on 4 branches.

2.2 Source Driving Circuit

The optical source driving system contains anode voltage generation using a linear dropout voltage regulator with 5 V output and cathode current sink circuit using an op-amp and a digital-to-analog converter. There are 2 independent anode voltage generation circuits and 4 independent cathode current sink circuits. These circuits combined with a MOSFET based 8 x 3 multiplexer allows for current control of 82 optical sources (Figure S8). Current for red sources can be controlled from off (0 mA) to 323 mA in steps of 1.43 mA and current for infrared sources can be controlled from off (0 mA) to 649 mA in steps of 2.72 mA. For safety reasons there is a 909 mA combined current limit for each anode voltage generation circuit. Once the current is set Spotlight can pulse sources on and off with a minimum on time / off time of 20 usec. Default operation of Spotlight uses 250 mA current setting for both red and infrared sources with 980 usec enable time for each source.

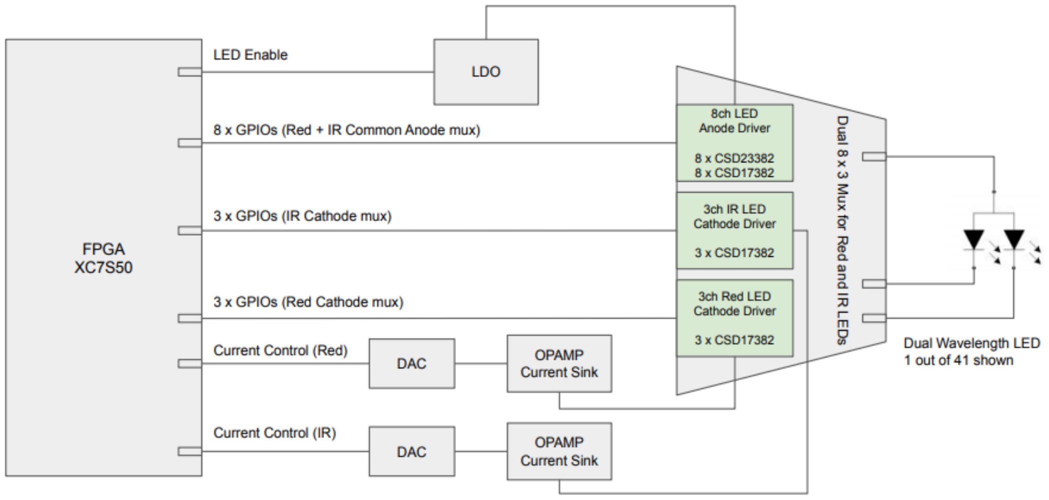

Figure S8. Source driving circuit diagram

2.3 Source optical power

The source optical power output and coupling efficiency was measured using a custom integrating sphere from Labsphere (P/N CSTM-LPMS-060-SL) with a large enough input port (2.75 inches) to accommodate a single module. The connections between hardware are shown in Figure S9. The measurements are conducted with 250 mA driving current for the LEDs.

The coupling efficiency is approximated with the LED source power taken from the manufacturer datasheet. The expected power without the ferrules is 150 mW, 115 mW for 680 nm, 850 nm respectively. The average power for LEDs measured at the ferrule output was 35.2 mW and 30.9 mW for 680 nm and 850 nm, resulting in coupling efficiency of 23.6 % and 24.8 %, respectively. The range seen in measured optical power among channels of a single module (3 mW and 10 mW for 680 nm and 850 nm, respectively) is largely due to changes in spatial location of the ferrule at the inlet of the integrating sphere. The measured power is less around the perimeter of the input port due to a higher number of internal reflections before reaching the integrating sphere detector.

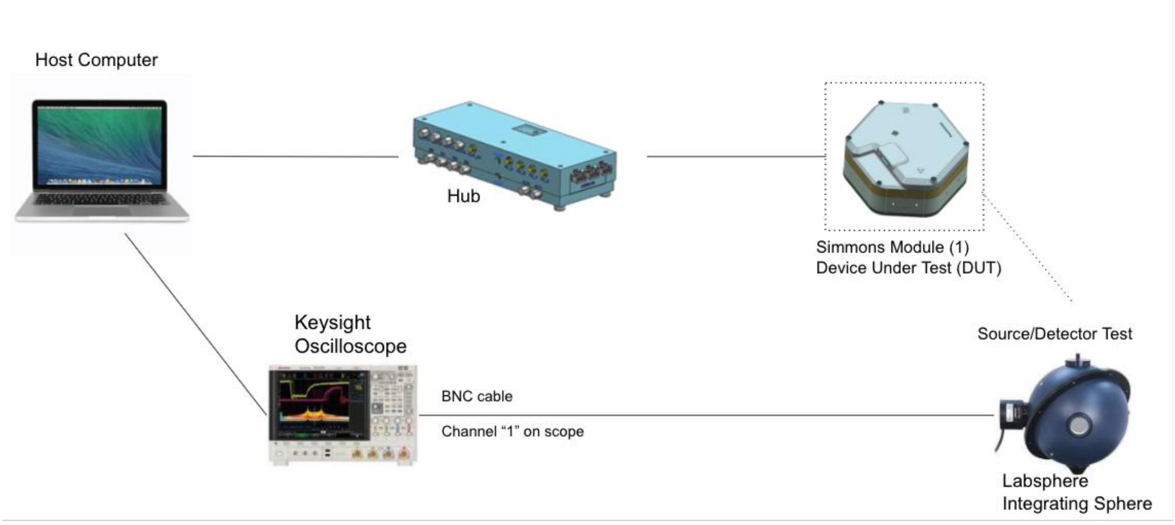

Figure S9. Overview of experiment setup using Labsphere integrating sphere.

2.4 Source Illumination Pattern

The source LEDs are multiplexed to enable acquisition from all the source-detector pairs without interference. The standard illumination pattern involves a 1 ms pulse width alternating between 680 nm and 850 nm wavelengths on the same LED. Between each pulse is a 1ms ambient frame, with a system effective duty cycle of 50 % (Figure S10). With 82 distinct steps, the cycle time of the module to illuminate all sources is 164 milliseconds resulting in a system frequency of 6.1 Hz (effective duty cycle per LED is 1ms/164ms ~ 0.61%).

The illumination pattern was designed to allow a sufficient recovery time of source adjacent SiPMs. The pattern operated with a circular pattern such that saturating nearby detectors have a longer time to recover before the next illumination cycle. As such, the illumination pattern appears to “sweep”, rotating around the center of the module.

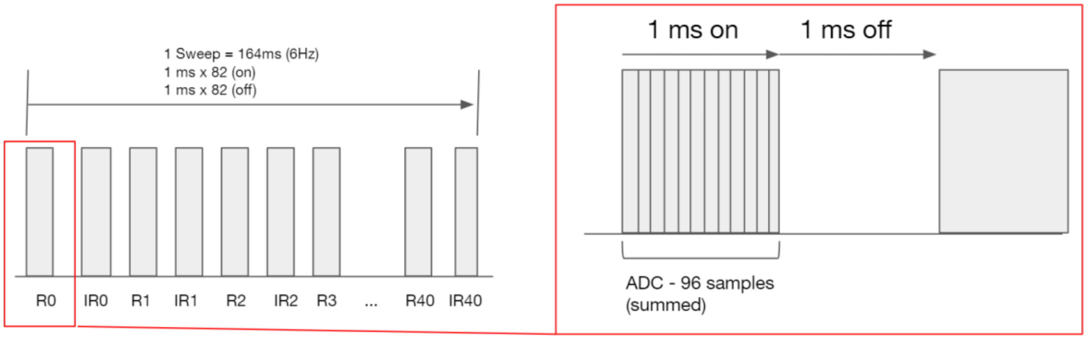

Figure S10. Source sequence diagram for one module.

2.5 Static phantom

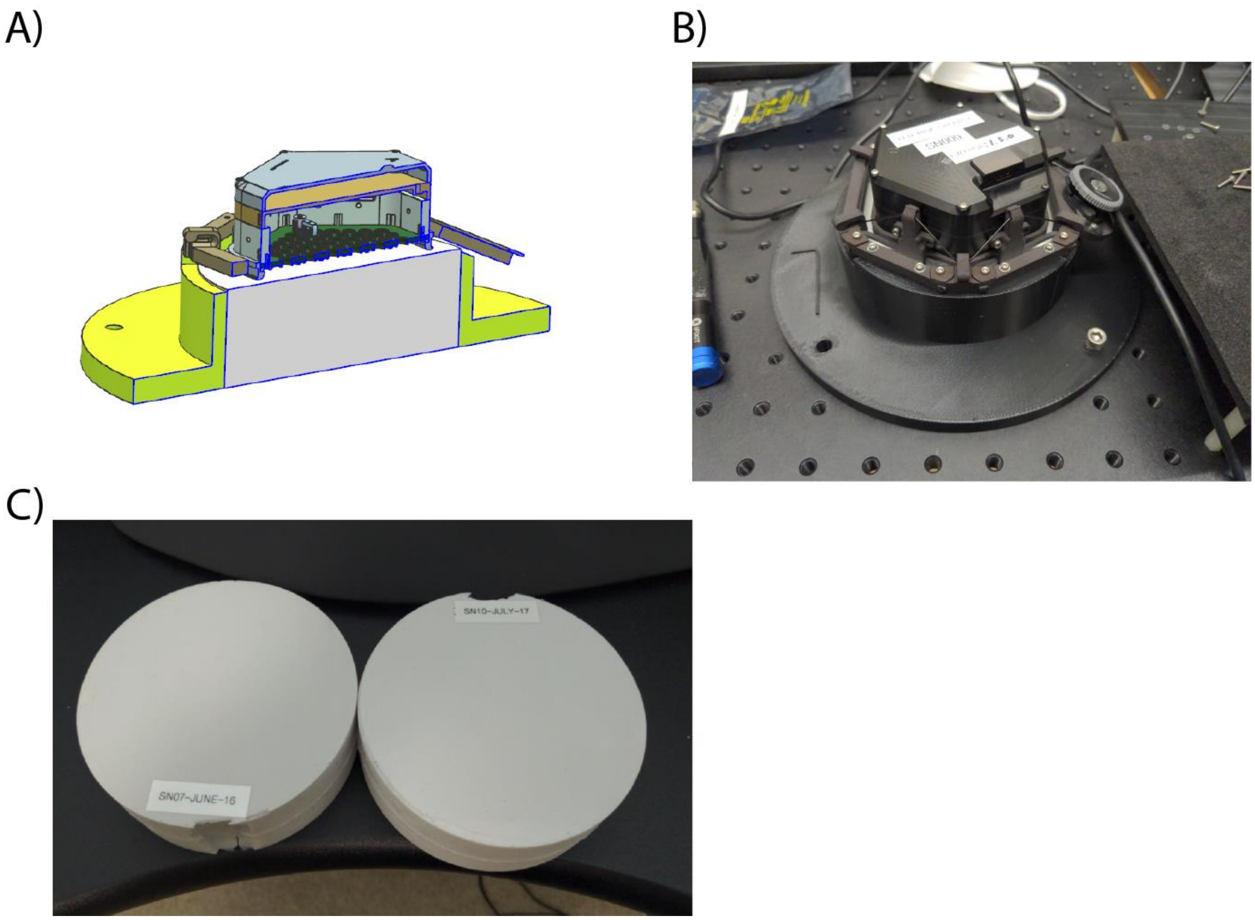

Figure S11. Static phantom setup for Spotlight module validation: A) Cad drawing of the test setup cross-section. White is the phantom material. Spotlight module is shown mounted on top. B) Phantom test setup. C) Cylindrical static phantoms.

2.6 Detector FPC and detector interface circuit

We designed a minimal analog front end circuit using just one transimpedance resistor of 4.99 kOhm, and carefully laid out the tracks with shielding on 5 layer FPC. 10 mil pitch of each signal was used to route the signals from each SiPM through the small width of the branches.

The detector FPCs are connected to the Mainboard through 40pin FPC connectors. The signals in the connectors consist of a bias voltage and raw voltage converted SiPM signals which are connected to the input of analog-to-digital converters. There are 2 bias voltage generators which provide bias for each detector FPC and each is capable of providing voltage between 20.4 V and 31.08 V in 41.7 mV steps. Each bias voltage generator includes a voltage boost IC (Maxim MAX1932) and linear dropout regulator (Texas Instruments TPS7A4001) and is capable of providing 8 mA. There are 5 x 8-channel ADCs providing simultaneous data from 39 SiPMs. Each ADC channel is capable of 18-bit data with maximum throughput of 500k samples per second. Default operation of the ADC is 100k samples per second along with 96x oversampling in FPGA (see Mainboard section).

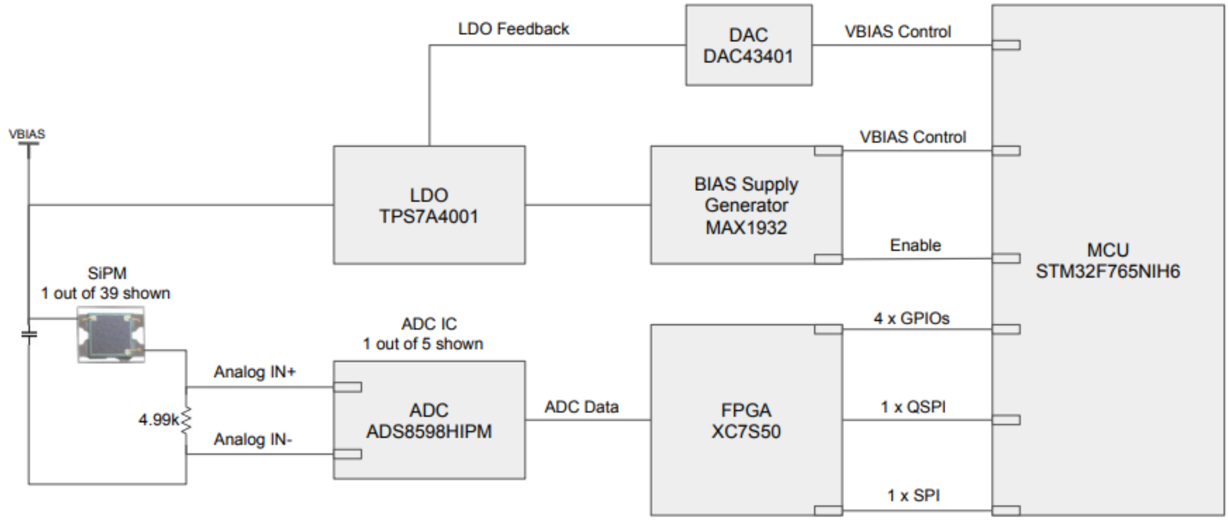

Figure S12. Detector interface circuit diagram.

2.7 Detector Performance

High SNR was achieved using high-performance SiPMs in surface mount small form factors. However, SiPM is known for its limited dynamic range because the number of different output voltage levels is determined by the number of microcells composing the device.The amplitude of the optical signal to be measured needs to cover from 10 fW to 1 uW, which translates to 160 dB of dynamic range. We were able to approach this target by adopting negative feedback on the bias voltage. As the SiPM signal becomes larger, the bias voltage gets reduced, which effectively increases the gain of the SiPM. This creates a non-linearity in the transfer function in the circuit, which we can correct after measurement. Figure S13 shows an example of light fall-off (LFO) curve from a static phantom measurement session, where the channel light levels have a log-linear fall-off versus distance, characteristic of photon diffusion through biological tissue. From this LFO we can derive the effective system dynamic range is roughly 120 dB, taking into account effects of the downstream digitization circuits.

For typical optical receivers, the pre-amplifier circuit needs to go inside the ferrule to prevent the sub micro ampere SiPM current signal from being overwhelmed by the interference signal at the FPC trace. Traditionally, transimpedance amplifiers are used to interface the photodetector and the rest of the conditioning circuit, but there isn't enough room inside the detector ferrule. Instead, we developed the interface circuit with just a passive SMD resistor and capacitor (0201 form-factor). The high gain of SiPM together with the high-value resistor resulted in a signal that was a few mV, enough to pass through the FPC without interference.

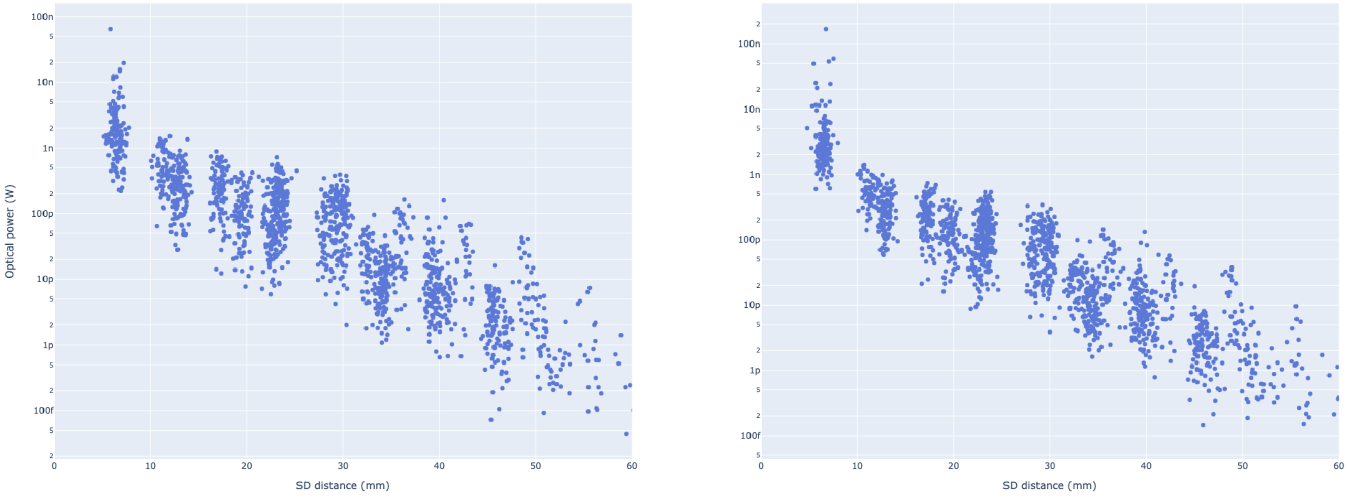

Figure S13. Light Fall Off (LFO) plot for one module (Left: 680nm, Right: 850nm) from phantom measurements. The x-axis represents source detector (SD) distances in mm in a single module. The y-axis represents Optical power in watts in logarithmic scale. Channels with the same SD-distance are plotted with a small jitter for visualization purposes.

2.8 Thermal Drift

During the process of data collection, we observed instances of thermal drift both in the laboratory and with human subjects, manifesting in slight rise in module temperature and monotonic increase in measured optical power. The rate of drift was gradual, plateaus after long operation (~hours) and could be effectively mitigated using filtering methods (detrending and bandpass, see section on Signal Preprocessing). The maximum of variation in optical power that has been observed over 1-minute periods was 0.5%.

2.9 ADC board and Main board

In addition to the FPC and optical ferrules, the module also contains the Mainboard (Figure S14) and ADC board (Figure S15) for signal acquisition and processing. The Mainboard contains a Xilinx Spartan-7 (XC7S50) FPGA and a STMicroelectronics STM32 (STM32F765NIH6) MCU which collectively interfaces with multiple DACs, ADCs and sensors for illuminating optical sources and reading measurements from optical detectors. The mainboard also has a USB-C port which provides a USB 2.0 connection to the host computer along with pulse per second (PPS) signals for time-synchronized operation of multiple module modules. The PPS signal is distributed via Spider – a USB-hub capable of communicating with 6 Spotlight modules. 82 individual optical sources are controlled by 4 sets of 8 x 3 multiplexer circuits implemented by 8 anode drivers and 3 cathode drivers. Simultaneous read from 39 optical detectors is achieved by 5 x 18-bit, 8-channel ADC ICs (Texas Instruments # ADS8598HIPM).

The components of the Mainboard (FPGA and MCU) connect to each other via 21x SPI port at 27 MHz, 1kHz ADC sampling, and 4x GPIOs. Collectively they provide control to various ADCs, DACs and sensors along with communication to a host computer over USB 2.0 interface.

The FPGA is responsible for the following tasks:

- Turning red and infrared source LEDs on and off with configured current, duration and sequence (shared between FPGA and MCU, MCU override values)
- Providing clock and start conversion signals for ADC chips and simultaneously receiving 18-bit data for 40 ADC channels at 100k samples per second.
- Accumulating / 96x oversampling ADC data for 39 channels and transferring it to MCU for streaming over USB 2.0 to the host computer. Streaming rate 1kHZ. (up to 4kHz was tested)
- Receive external clock for synchronization of source enable / disable and ADC start conversion so that multiple Spotlight modules connected to single Spider can stay synchronized.
- Safety shutdown of red and infrared source LEDs in case of fault

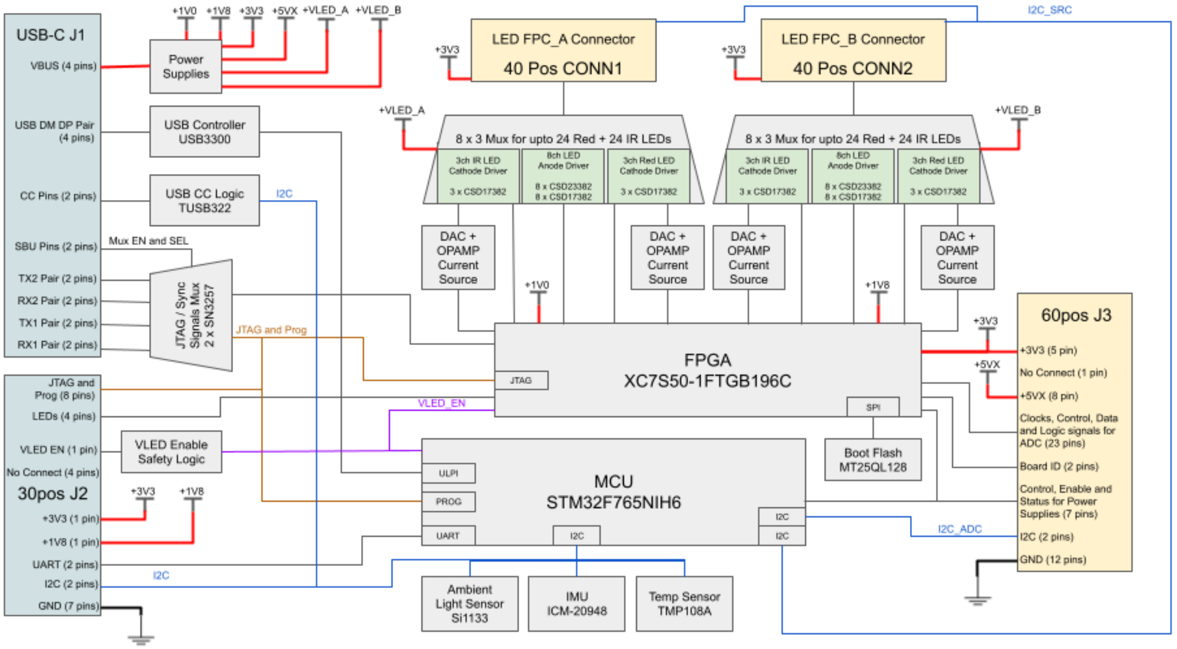

Figure S14. Main board block diagram.

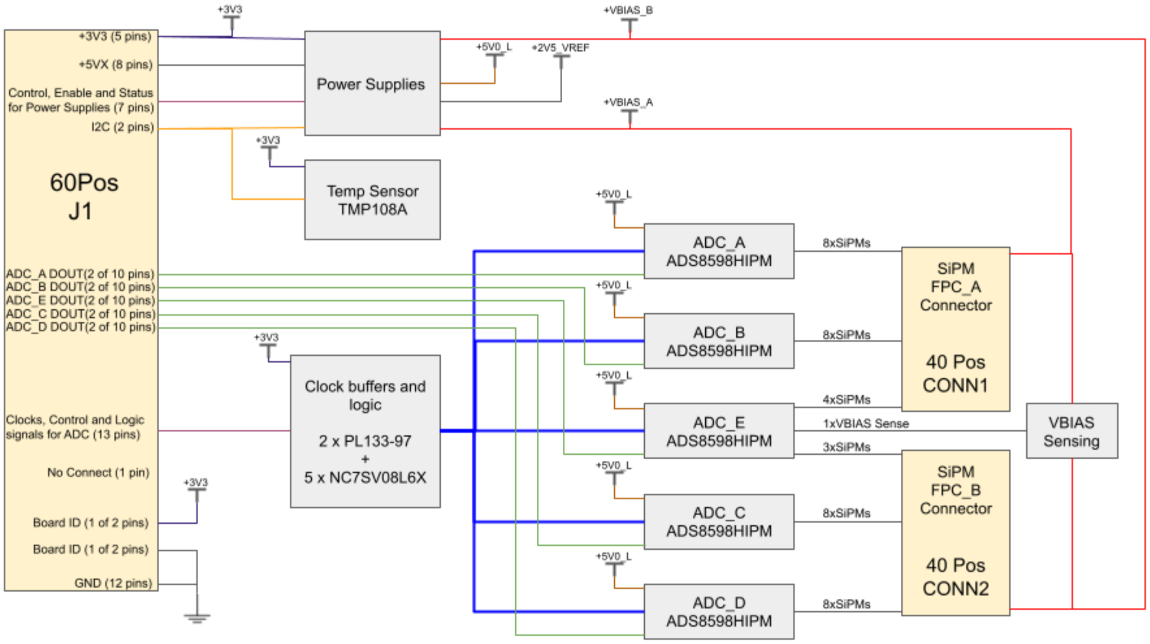

Figure S15. ADC board block diagram.

2.10 ADC signal processing

Every millisecond, the Xilinx Spartan-7 will accumulate and aggregate 96 samples (Figure S10) for each detector and will interrupt the STM32F765 to notify new data is ready to be retrieved. The STM32F765 will read the aggregated data for each 39 detectors in a single SPI transaction and send it to the host PC over USB without modifying the data. It is the responsibility of the host computer to read the data in the allocated time or queue up USB transfers. A library such as libusb can be used to read the bulk transfer endpoint from the device.

2.11 Sensors data packet

Sensor data is synchronized to the ADC data and sent every 4 milliseconds. The sensor data consists of two temperature sensor readings, an ambient light sensor, a power monitor and an inertial measurement unit (accelerometer, gyroscope and includes a magnetometer). A library such as hidapi can be used to read sensor data over HID reports.

2.12 Synchronization across Spotlight units

The goal is for Spotlight units to capture data at the same rate (frequency) and at the exact same time (identical phase). In order to accomplish this goal, a single clock is shared from the Spider hub to each Spotlight FPGA unit, driving data capture and data transfer. In order to align individual samples, a monotonically increasing counter is assigned to each ADC data capture. As each unit will receive the first clock cycle at the same instant, and further clock cycles are also received at the same instant by all units, the frame counters will be in perfect sync between modules.

3. System Firmware and Host Software

3.1 Identifying a Spotlight unit

During assembly, each Spotlight unit is assigned a unique serial number. This serial number is written to an area of the microcontroller non-volatile flash memory. The same serial number is hand-written on the physical unit so it can be easily identified. To identify the physical location of the unit, a user edits a configuration file before running an experiment, where the expected serial number is matched to physical locations on the head. This file is read at runtime and verifies all Spotlight units (up to six) are connected to the host PC by querying each unit serial number. Each USB data stream is mapped to the serial number so any data received can be identified to a location on the head.

3.2 Describing a Spotlight unit

A single configuration file describes the physical properties of the system. This file describes the physical layout of each Spotlight unit, which include the coordinates (in 3-D space) of each detector and source. This file also includes distance and rotation of one Spotlight unit in relation to the other, which describes precisely where units are located on the head.

3.3 USB communication

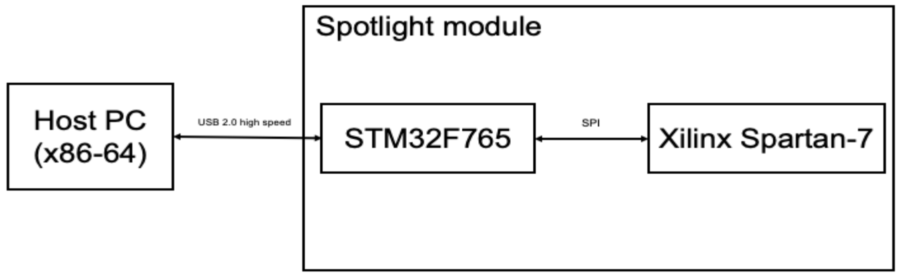

Figure S16. Spotlight SW module interface.

A Spotlight module requires a USB connection to a host computer for data and power. Spotlight module will establish a USB 2.0 high speed connection to the host computer to send data and receive commands. The module connects to a host computer either directly via USB Type-C to Type-A or by connecting into our custom USB hub (Spider).

The device exposes 1 USB configuration, with 4 USB interfaces. The first two interfaces that enumerate are USB CDC/ACM. The purpose of USB CDC/ACM is to expose any firmware logs. The third interface contains a single IN bulk endpoint. This endpoint is used to send the ADC data at 1kHz. The last and fourth interface is USB Human Interface Device (HID) with a single IN endpoint. This endpoint transfers sensor data (temperature sensors, ambient light sensor, power monitor, and inertial measurement unit) at 250 Hz. The HID interface also allows feature reports to be sent to the device to execute specific commands (such as starting or stopping the source LEDs).

3.4 Firmware and Host Software

Firmware is responsible for following tasks:

- Interfacing with host computer over USB 2.0 high speed interface
- Setting bias voltage of SiPM through LDO and SMPS power supply
- Configure ADC sample rate, source pulse rate and oversampling
- Turning red and infrared LEDs on or off at the request of the host computer
- Receiving ADC data for 39 channels from FPGA and forwarding it to host computer via high speed USB
- Streaming over USB data from temperature sensors, ambient light sensor, power monitor and inertial measurement sensor in Spotlight system.

FPGA is responsible for following tasks:

- Generating LED illumination pattern
- Providing LED safety logic which triggers when duty cycle is high and shut-off automatically
- Receiving 5 ADC output signal and packing into FIFO readable by MCU
- Dual switching FIFO to swap reading and writing operation between ADC and MCU

Host software is responsible for following tasks:

- Identifying a Spotlight unit
- Describing a Spotlight unit
- Synchronizing across Spotlight units
- USB communication
- ADC signal processing
- Sensor data processing
- Transferring illumination pattern to FPGA

A Spotlight unit is unaware of the number of other units streaming data in parallel. A unit is also unaware of its physical location on a user's head. In order to perform correct analysis and decoding, it is necessary to identify samples that were taken at the time. We also need to identify where each Spotlight unit is located on the head.

4. Signal Preprocessing

1. Bad channel removal: Due to less than 100 % yield of the optodes, each Spotlight module can have a few bad source and/or detector optodes. These bad optodes would result in power measurement values less than or equal to 0 (as system noise level is never 0)
    a. Offline processing: Any channels with such anomalous measurements are discarded.
    b. Real time processing: Channels of the incoming sample with non-positive measurements are replaced with their respective last positive-valued sample measurement. If the first sample of a channel is non-negative, its value is set to 1 such that its optical density value is equal to 0 (see step 3).
2. Detrend: For offline processing, linear trend/drift is removed from each channel's measurement time series by subtracting from it the best linear fit as a function of time, then offsetting the resulting time series by the temporal mean of the original data. This step primarily removes sensor drifts due to thermal effects (see supplementary, Thermal Drift). The mean offset procedure ensures the resulting values are positive and valid for the subsequent optical density calculation. Any negative values remaining after the offsetting procedure (less than 10% of time points per channel) are interpolated by the surrounding non-negative values.
3. **3.** Optical Density Conversion: Concentration changes of the hemoglobin species during cortical activation result in changes in light absorption relative to baseline (resting state) [57]. We estimate this change in light absorption, referred to as delta optical density, ΔOD as the following. Note that in these calculations, ΔOD of 0 would indicate a lack of cortical activity deviating from the resting state.
    a. Offline processing: $-\,log\,(\frac{\Phi(t)}{\bar{\Phi}(t)})$, where $\Phi(t)$is the power measurement time series, and $\bar{\Phi}(t)$is its temporal mean and relative baseline measurement [58]
    b. Real time processing: $-\,log\,(\Phi(t))$.
4. **4.** Bandpass Filtering: Bandpass filtering is applied to the optical density (OD) time series to remove unwanted frequency contents (e.g. heartbeat, respiration, Mayer wave) [59] between 0.01 and 0.1 Hz.
    a. Offline processing: A 5th order butterworth bandpass filter with cutoff frequencies at 0.01 and 0.1 Hz is applied forward and backward in time to achieve zero-phase filtering.
    b. Online processing: To minimize filtering-related group delays in causal filtering, a 5th-order high pass butterworth filter with cutoff frequency at 0.01 Hz is cascaded with 3 first-order low pass butterworth filters with cutoff frequencies staggered at 0.80 Hz, 0.37 Hz, and 0.62 Hz. These filter specifications were determined from parameter sweeps to optimize decoding accuracy while minimizing filtering latency.
5. **5.** Superficial regression: Superficial regression [27,60,61] is applied to remove physiological artifacts from longer (SD-distance) channels using measurements from shorter channels. A linear adaptive noise canceling (ANC) approach [62] is applied to each Spotlight module, where noise calculated from channels within SD-distance range of [0, 15] mm is removed from "signal" channels with SD-distance above 15 mm. This noise removal process is done separately for groups of signal channels within successive 10mm SD-distance ranges. This

step discards the shallow channels and yields only the cleaned signal channel measurements.

a. Offline processing: The noise is calculated from taking the average of all channels in a given module and within the specified distance range, then taking its temporal zscore. Each signal channel is converted to its temporal zscores. The single noise time series and the signal measurements then enter into the ANC algorithm.
b. Online processing: Differ from offline processing in that zscoring is not conducted on either noise or signal channels. The ANC parameters were determined from parameter sweeps to optimize decoding accuracy while minimizing latency.

6. Conversion to hemoglobin: To generate evoked response in Figure.2B (in the main manuscript), the superficially regressed channel measurements are converted to values proportional to oxyhemoglobin (HbO) and deoxyhemoglobin (HbR) concentration through modified Beer-Lambert Law [57,58,63]. The extinction coefficients for 680 nm and 850 nm light were interpolated from the data compiled by Scott Prahl [64]. The differential path lengths (DPF) for these wavelengths (λ) were calculated assuming age of 30 using the relationship derived in [65]:

$$DPF = 223.3 + 0.05624 \times age^{0.8493} - 5.732 \times 10^{-7}\lambda^3 + 0.001245\lambda^2 - 0.9025\lambda \quad (Eq.1.)$$

5. Analysis metrics

5.1 Pulse SNR via CFAR

For each PSD, CFAR is used to determine the presence and location of a pulse peak (frequency range from 0.5 to 2 Hz). CFAR is an adaptive peak finding method taking into account the noise level at each frequency bin, and requires a peak to be above a threshold that is some multiple of the local noise level to be counted. This threshold is set by the false-alarm rate. If multiple peaks are detected, the frequency bin with the highest magnitude would be selected.

The pulse SNR is calculated for all channels that CFAR determines to have a peak in the pulse range (and set to 0 otherwise). This value is 10 * log10(signal / noise), where signal is the sum of n + 1 frequency bins immediately surrounding the peak, divided by the sum of *n* frequency bins within a train bandwidth surrounding the guard bandwidth (see Figure **S17**). n is chosen to be the minimum of train and guard bandwidth. Pulse SNR less than 1 is also set to 0.

The CFAR parameters: guard bandwidth, train bandwidth, false alarm rate were determined from parameter sweeps on pilot datasets. For this pilot dataset, the mean PSD for short (<= 7 mm), medium (25 to 55 mm), and long channels (> 55 mm) were calculated, and the presence of a pulse peak was manually labeled. The parameter sweep then searched for the CFAR parameter set that maximized pulse peak detection matching the manual labels.

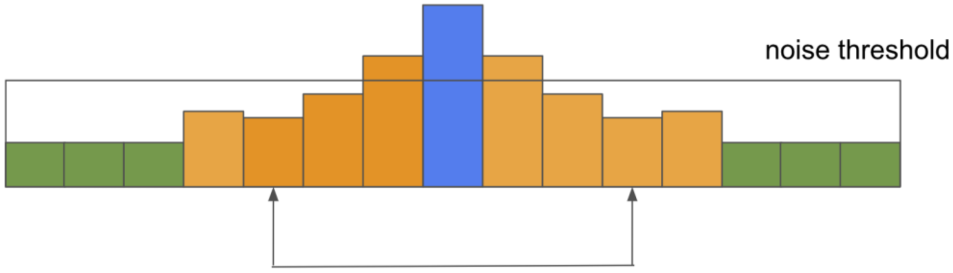

Figure S17. Illustration of CFAR pulse SNR. The blue bar is the frequency bin under test. The orange bars are the guard bins with guard bandwidth of 8. The green bars are the training bins with train bandwidth of 6. The horizontal black line indicates the noise threshold the bin under test's magnitude needs to reach to be considered a peak. To calculate pulse SNR, the sum of frequency bins between the two arrows is equal to the *signal* term, and the sum of all green bins is equal to the *noise* term.

5.2 Characterizing functional activation with general linear model (GLM)

GLMs [66] were used to characterize the functional activation by the participant's finger movements. The experiment conditions of "Left" and "Right" hand tapping are encoded as two separate "box-car" time series that are the same length as the optical data, with value set to one during time points corresponding to their respective trial periods, and zero otherwise. A null condition trial is then represented as period of zeros in both box-car time series. In offline analysis, the start and the end of a trial was determined by the first and last event of the correct key presses logged in the experiment. In real-time decoding, the start and the end of a trial was estimated based on the visual cue presentation time. The "box-car" representation of each condition was then convolved with the canonical hemodynamic response function (HRF) [67–69], which represented the "expected hemodynamic responses" assumed in this model and served as regressors in the GLM (denoted as ${s_t}^{left-hand}$ for the "left hand" condition at time t for example).

For each valid channel (or source-detector pair) i, the preprocessed optical data (${y_t}^i$) was linearly regressed against these regressors ($y \sim \beta^{left-hand}{s_t}^{left-hand} + \beta^{right-hand}{s_t}^{right-hand} + constant$), and the linear coefficient for each condition (e.g. $\beta^{left-hand}$) was estimated via ordinary least squares. Note that in the GLM analysis, only the time points within the valid trial segments were considered, the inter-block time points and inter-trial intervals outside the segments and invalid trials were removed.

The r-squared of this linear regression evaluates the percentage of variance explained by the expected hemodynamic response for all the conditions, and thus can be used to characterize how strong functional responses are for each channel. We additionally tested whether the regression coefficients are significantly non-zero by computing the t-statistics (estimated coefficient value divided by the estimated standard deviation of the coefficient, e.g. $\beta^{left-hand}$ / std($\beta^{left-hand}$)), and then applying z-tests (Wald test) to obtain the p-values, assuming a Gaussian null distribution with our relatively large sample size.

As we cannot assume that the optical data are independently and identically distributed (i.i.d) over time, we applied a bootstrap procedure [70] before applying the z-test on regression coefficients: trials were sampled with replacement $n_{bootstrap}$ times independently, and a GLM was fitted for each of the resampled set, yielding $n_{bootstrap}$ estimates of coefficients, across which the coefficient standard deviations were computed. ($n_{bootstrap}$ = 200). Similarly, we can measure a channel's differential activation between conditions by testing whether the difference of the regression coefficients ("contrast" between conditions), e.g. ($\beta^{left-hand} - \beta^{right-hand}$), is non-zero.

After the p-values of Z-tests for either the coefficients or contrasts were obtained, false discovery rate (FDR) correction [71] implemented in MNE-Python [72] was used to correct for multiple comparisons over all channels.

5.3 Arch Metric

The arch-metric provides a more fine-grained way to quantify and compare overall hemodynamic content captured by the system compared to simpler metrics without taking into account channel SD-distance (i.e. mean/median of all R2 values). As channel SD-distance increases above 20 mm, the measured signals should become more sensitive to cortical activities. Setting $m(d_1, d_2)$ as baseline and summing additional increases in the subsequent SD-distance ranges reflect this property. In Figure S18, only significant R2 values above each 5 mm SD-distance range's mean-R2 threshold are plotted for each subject. Even without looking at the y-axis values, the "arches" formed by the bottom of each column of R2 points give indications of different sessions' hemodynamic content.

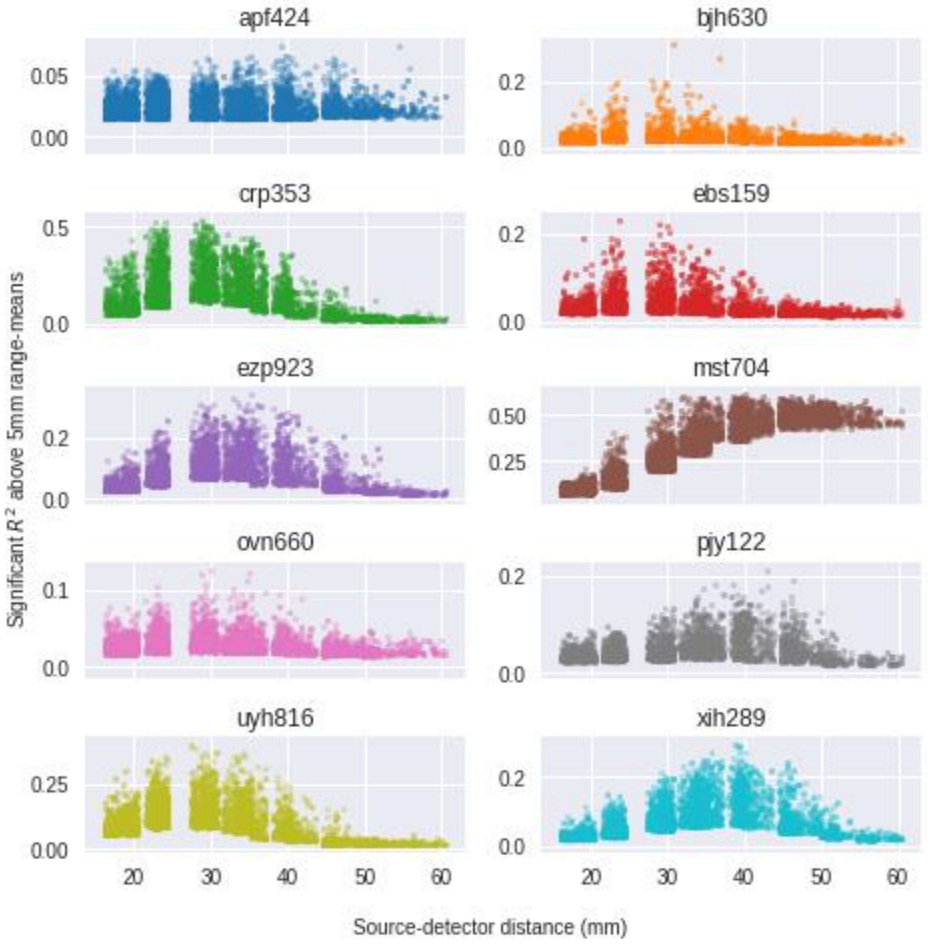

Figure S18. Source-detector distance vs. thresholded GLM r-squared (R2) values for different subjects. For each 5mm SD-distance range, the mean R2 value is calculated and only R2 values greater than that threshold are shown. Each subplot overlays all sessions for each subject (dots with different transparency). Only significant R2 values are included.

6. Task Condition Decoding

The following are the steps for decoding the task conditions based on the hemodynamic activities.

6.1. Segmenting optical data into trials

Let 0 be the onset of a trial (defined as the onset of the visual cue), the time series of preprocessed optical data from -5 to 20 seconds were used for offline decoding. The same segmenting procedure was applied to all channels and all trials. The segmented data was further normalized to a range of 0 to 1 in the decoding analysis of the results section.

For a real-time decoding, time series of preprocessed optical data corresponding to from 0 to 15 seconds were used so that feedback can be provided within the 20 seconds trial length.

6.2. Channel ranking and selection

For each channel, the t-statistics of the coefficient for each condition (except for "Null") and pairwise contrast between conditions (except for "Null") were computed using the aforementioned GLM procedure. For each condition and pairwise contrast, we sorted the absolute values of the t-statistics and selected the top n_0 channels, then we took the union of them as the channel set for decoding. The n_0 channels of all the conditions and contrast considered may overlap; we denote the final number of the union of channels as $n_{channel}$.

The t-statistics for ranking channels were computed using an analytical solution assuming i.i.d samples, which were not accurate for statistical testing; however, in preliminary analysis on pilot data, we observed that these t-statistics were highly correlated with the bootstrapped version and requires much shorter computation time. Since we are mainly ranking and selecting channels rather than hypothesis testing, we used the analytical solution to reduce computation time.

6.3. Predicting hemodynamic response with multi-channel data

Based on the condition label (e.g. "Left hand", "Right hand" and "Null"), we created a "hemodynamic response template" for each trial, of size $T \times n_{condition}$, where T denotes the number of time points in the segmented trial data, and $n_{condition}$ denotes the number of conditions. The column corresponding to the target condition consisted of a canonical hemodynamic response function (HRF) with respect to the expected onset (a real-time decoding) or actual key press onset (offline decoding); and the remaining columns of non-target condition were set to zero.

During training time, we learned a linear regression from the segmented optical data of selected channels (**X** of size $(n_{trial} \times T) \times n_{channel}$) to the hemodynamic response template (**Y** of size $(n_{trial} \times T) \times n_{condition}$) with $n_{trial} \times T$ samples, where the linear coefficients needed are of size $(n_{channel} \times n_{condition})$. Given a penalization parameter, the linear regression model was penalized on the L1 norm ("lasso regression"). At testing time, the linear model learned during training was applied on data from testing trials to obtain the prediction of the hemodynamic response template $\hat{Y}$.

6.4. Classification

Given the predicted hemodynamic response template output by the linear regression model, we computed the similarity between the prediction $\hat{Y}$ and the true hemodynamic response template Y, as the dot product of the two time series for each condition. The dot product for all conditions were then fed into a linear discriminant analysis classifier to classify the condition labels.

6.5. Selection of hyperparameters

A cross-validation approach was used to select the hyperparameters ($n_{channel}$, regularization parameters, etc) from the training data, and the hyperparameters that yielded the highest decoding accuracy were selected to train a final model that was applied on the testing data.

6.6. Permutation test

Note that the class labels in some experiments were not balanced (e.g., roughly 40 % Left Hand, 40 % Right Hand and 20 % Null). To obtain an estimation of the chance level, we introduced a permutation test. After hyperparameter selection, the regression model and classifiers are trained. The same trained model was then applied on each of the permuted instances of testing trials, where the condition labels in the testing trials were permuted 200 times. The mean accuracy across the permutation was obtained as a representation of the chance level.

6.7. Train-test splits

For offline single-session decoding, we used the first half of the trials as training data and the remaining half as testing data, which simulated the close-loop experiment in which we trained the models with some previously collected data and tested on the subsequent data coming in.

For real-time decoding, a decoder as described above was trained with two blocks of trials without feedback, and the trained decoder was then applied to provide real time feedback for subsequent testing trials.

7. Differentiating individual digits of the same hand (Digit discrimination)

Previous fMRI studies demonstrated a topography representation of individual digits in the primary sensory cortex, where representation of individual fingers is spatially apart. The separation can be 4-18 mm. Therefore decoding activation in individual digits can serve as a functional test of spatial resolution of our system. Few fNIRS or DOT studies had looked into this. Notably, Habermehal et al, 2012 [73] visualized the spatial activation when thumb and pinky fingers were stimulated by vibrotactile stimulus and compared the activation map after tomographic reconstruction with that from fMRI. However, they did not report the discriminability between fingers.

To further evaluate the SNR of the neural signals, specifically evaluating the ability to discriminate between individual digits within a single hemisphere / optode array, we conducted an additional "digit localization experiment".

7.1 Digit discrimination: Experimental protocol

Two sessions were collected in a single day -- one session involving the left hand and one involving the right hand (with hand order counterbalanced across participants). When the stimulus displayed a hand cue, participants tapped the highlighted finger until the cue disappeared (6 second duration). Participants remained at rest during the null condition and also during the post-stimulus interval (post) period (20 seconds in duration). There were 3 conditions total per session: right (left) thumb, right (left) pinky, and null. Twelve trials were collected per block and 4 blocks were conducted (48 trials in total with 12 trials for each condition). Consecutive null trials were not allowed (to allow for a more engaging experiment). Each block was preceded and followed by 20 additional seconds of rest. Experiment duration was roughly 30 minutes long. The experimental paradigm is shown in the Figure S19.

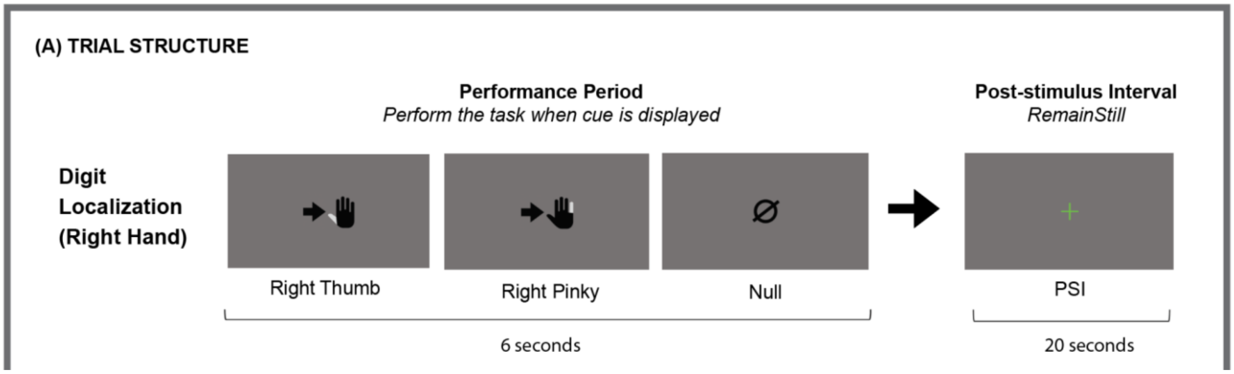

Figure S19. Illustration of the trial structure of the Digit Location Experiment: six seconds of performance period while visual cue being displayed was followed by a 20-second post-stimulus interval (PSI). The figure illustrates just the right hand for simplicity, but data were collected with both left and right finger-tapping.

7.2 Digits Discrimination: Analysis methods

Preprocessing, general linear models and decoding analysis are described in Materials and Methods; the same parameters were applied to both the hemispheric lateralization experiment and digit localization experiment.

In addition to analysis in the source-detector space, we also applied a very naive tomography model -- an infinite slab tomography model --- so that we could visualize the activation pattern in a spatially regular "voxel space" underneath the optode array. This model assumes an infinite plane at the optode interface, and homogeneous medium under the plane, where temporal changes of mu_a in pre-defined voxels in the medium below the surface accounted for the temporal variation of observations in the source-detector space. Note that this model is oversimplified, and we did not intend to use it as tomographic reconstruction, but rather just a spatial transform --- representing the data from interlacing source-detector pairs in a regular 3D space, such that potential spatial separations may be easily visualized. Mu_a of the medium was set to 0.01 mm^-1, and mu_s_prime was set to 1.0 mm^-1. Voxels of size 3 x 3 x 3 mm spanning 60 x 60 mm area and from 18 mm to 12 mm under the boundary plane (20 x 20 x 3 voxels in total) were defined in the forward model, and a regularization of L2 penalty of 1.0 was used in computing the inverse solution. The transformed data was further projected into the chromophore space as concentrations of HbO and HbR.

The t-statistics of the general linear model (assuming independent and identical residuals) for each voxel was computed and visualized, and the decoding pipeline with PCA of selected channels was used to distinguish the condition labels ("pinky" vs "thumb" of the same hand) in a half-split train-test paradigm for each session.

To further examine whether the multi-dimensional distribution of activation patterns between individual digits were different, a non-parametric permutation test with the minimum max discrepancy (MMD) statistic was implemented. Intuitively, this MMD statistic contrasts the within-class similarity versus between-class similarity. We first computed the Pearson's correlation of the single trial time series of a voxel/channel with the template hemodynamic response, obtaining an n_trials by n_channels (n_voxels) matrix for trials that corresponded to

tapping thumb finger or pinky fingers, then computed MMD on these correlation values. Permuted counter parts were obtained by permuting the class labels (thumb or pinky). The p-value was computed as the percentage where the permuted MMD values were greater than the original MMD values.

After obtaining the individual p-values, we combine the p-values using Fisher's method, implemented as `scipy.stats.combine_pvalues` in the scipy python package. The combining procedure tests a null hypothesis of several independent tests (for individual participants). If the combined p-value is small enough, then we have statistical evidence that at least for some participants in our experiment, the activation pattern of individual digits is different.

7.3 Digits Discrimination: Visualization of activation in channel space

Figure S20 shows the visualization of the GLM statistics: the summary statistics for each HbO channel on the activation of each condition in the GLM analysis (regression coefficient of the template hemodynamic response divided by its estimated standard deviation) for one example participant. As expected, the stronger activation (indicated by large positive t-statistics) for tapping the right thumb finger and pinky finger are both on the left module; however, in this visualization, as the haystack lines occlude each other, it was hard to see whether the activation pattern of the left modules appear different between the two fingers.

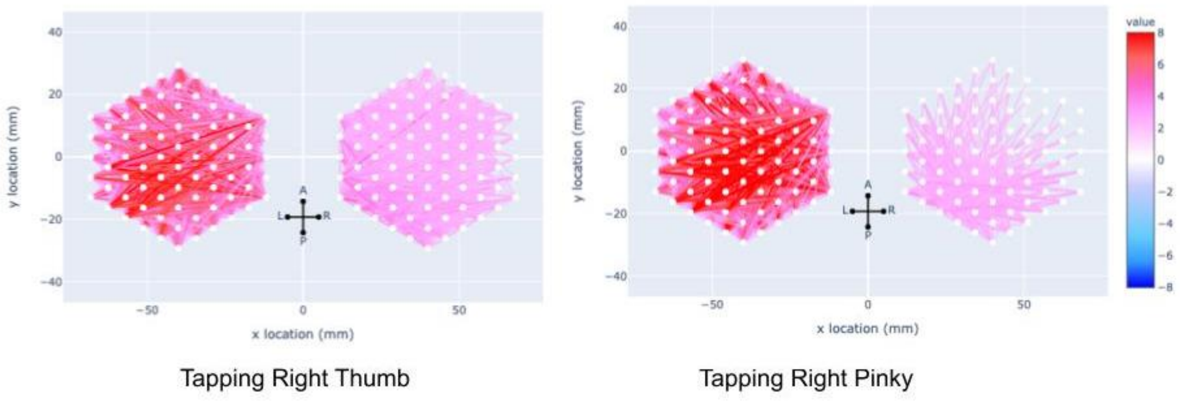

Figure S20. Haystack visualization of the GLM statistics (with bootstrapping) for one example participant (same subject as the one in

7.4 Digits Discrimination: Visualization of activation using an infinite-slab model

To circumvent the occlusion of the haystack visualization, we transformed the channel space data into a "voxel space", using an over simplifying infinite slab model. Instead of doing tomography, this step was more like just a spatial transform such that we can visualize the projection of activation pattern on a regular 3D grid.

Figure S21 demonstrates the visualization of the activation pattern of individual fingers (right thumb and right pinky) in the "voxel space" reconstructed using the infinite-slab model, from two participants. The color shows the GLM statistics of the corresponding fingers. For some participants, the GLM statistic showed the correct sign of activation (positive compared with

null) and was stronger on the expected side (in the voxel space underneath the left modules), as shown in

For some participants, the sign of activation seemed to shift from negative to positive as in Figure S22, although larger absolute values of the activation still seemed to be on the expected side (left modules). This may indicate that the tomography model we apply here is too naive to obtain the correct underlying voxel representation.

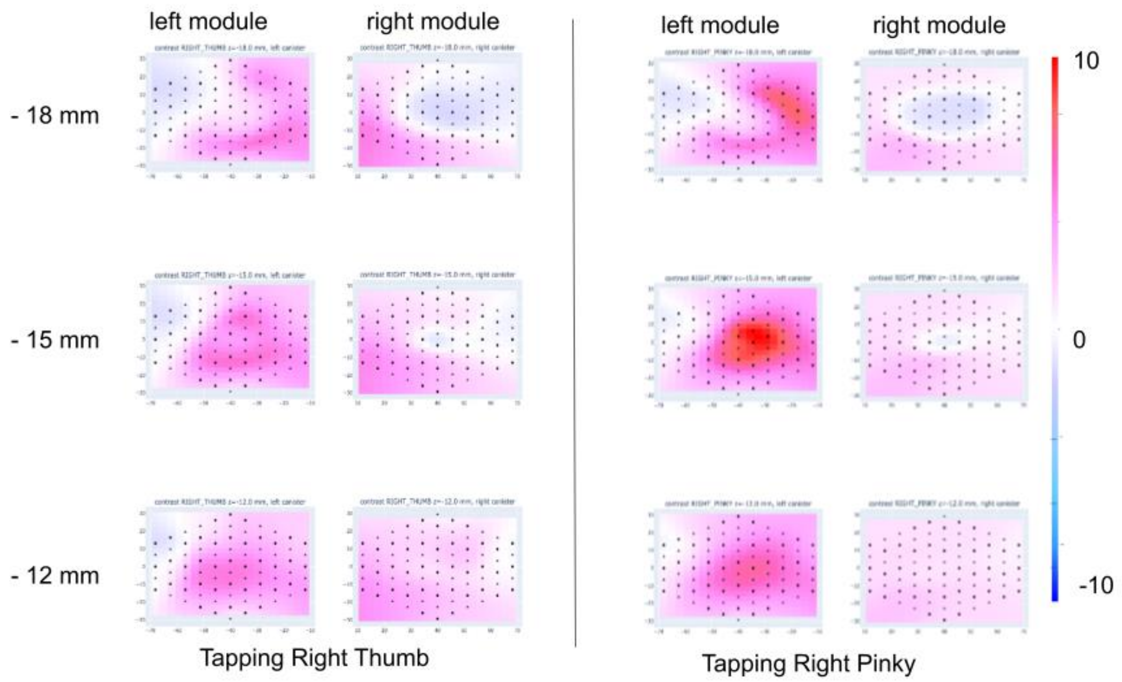

Figure S21. One example participant, for HbO, tapping right thumb and right pinky fingers.

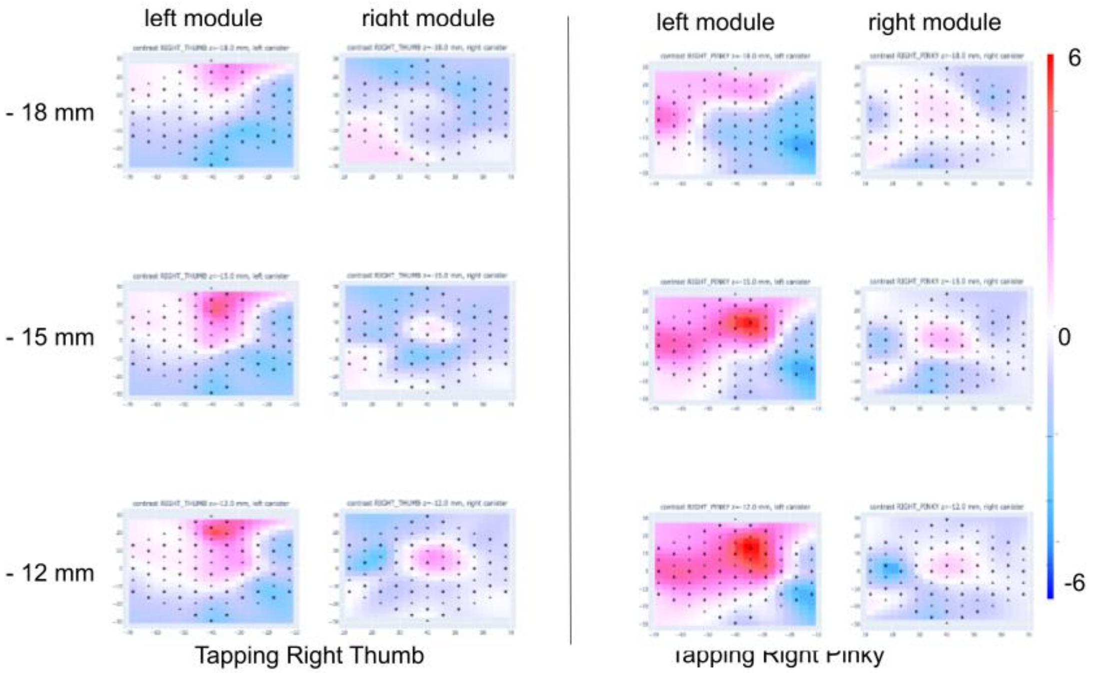

Figure S22. Another example participant, for HbO, tapping right thumb and right pinky fingers. Visualization of the GLM t-statistics (analytical solution, without bootstrapping) in the infinite slab voxel space

7.5 Digits Discrimination: Analysis of differentiating activation for different fingers of the same hand

Figure S3 shows the results from the decoding analysis in distinguishing between the two digits (thumb and pinky fingers) of the same hand, using either channel space data or "voxel"-space data reconstructed from the infinite-slab model. We did not observe a significantly larger accuracy than the 50 % chance level --- neither from a t-test of the mean accuracy across participants nor from combining p-values from binomial tests for individual participants.

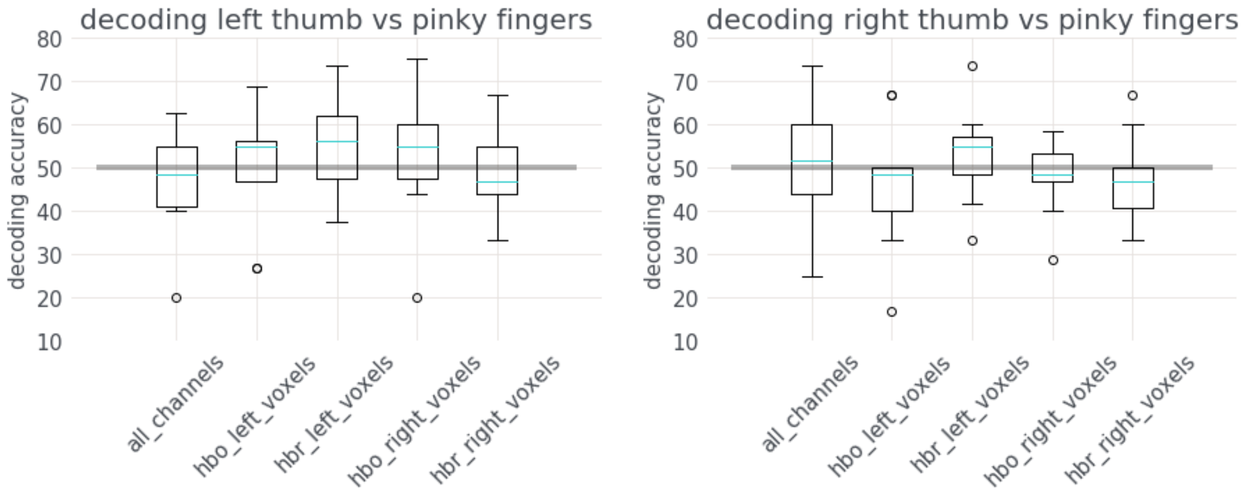

Figure S23. Accuracy of decoding individual digits (thumb vs pinky fingers) of the same hand, using channel space and "voxel space" data. Grey line at 50 % indicates the chance level. Green line indicates the median, box boundaries are quartiles.

We also ran a non-parametric permutation test using the minimum max discrepancy statistics, which describes the difference between within-class similarity and between class similarity of multiple dimensional data. Figure S24 included box plots of the log10 of the p-values from the permutation tests, using either all channel data or the voxel-space data. Red triangles indicate the combined p-values (not corrected for multiple comparisons) across all participants, which test against the hypothesis that in no participants we observe discriminability between activation of two digits. Only the combined p-value for the HBO signals in the right voxel space underneath the right module showed some marginal significance (p= 0.05) for distinguishing between left thumb and pinky fingers, indicating in at least some participants, the activation patterns in those voxels may be distributed differently between tapping left thumb and left pinky fingers. However, this may not survive a correction for multiple comparisons; we did not observe significant differentiability in other tests either --- we did not observe strong evidence of functional discriminability between individual digits in our experiments.

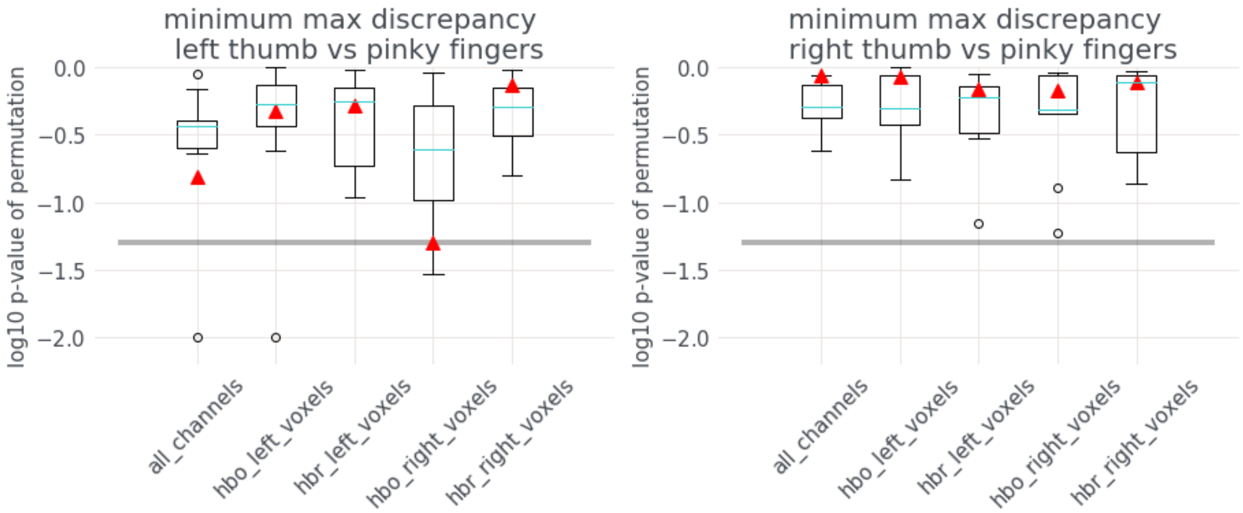

Figure S24. P-values of the minimum max discrepancy (MMD) permutation analysis. Box plot depicts the distribution of log10 (p-values) of individual participants, where the cyan lines indicate the medians and the box boundaries show quartiles. The gray line represents a reference point of $p = 0.05$. The red triangles indicate the combined p-values.

8. Localization Experiment

8.1 Localization: Motivation

The localization experiment demonstrates the advantage of placing the optode array directly over the region of interest. We achieved repeatable placement of the modules over the hand-motor cortex using custom-design self-donnable caps based on participant magnetic resonance imaging (MRI) scans. Prior to using the personalized self-donnable caps, the BCI cap fitting process was based on the continuous proportional coordinate (CPC) system in a manual process that allowed more room for error. This scenario involved the additional challenge of cap comfort, with only one cap with many knobs to accommodate all participants. The novel self-donnable cap design is based on individual MRI and functional MRI (fMRI) data where possible — MRI data to optimize ergonomics, and fMRI data to determine optode placement. The design also enables participants to don the cap without an in-person Research Assistant present, and to do so in a consistent way. We tested the performance of non-centered devices using an adjustable localization cap with three offset positions on each of the left and right sides, where the module was offset 25 mm from the centroid position in the anterior, posterior, and inferior directions.

Optimal centered locations for the study were found using fMRI, but not all participants have fMRI data. We've shown with this experiment that by utilizing a localization protocol, participants would be able to determine the optimal location by solely using the module. Visualized hot-spots give instructions to adjustments needed to find the optimally centered localization. The neural results obtained when the module is centered over the region of interest (compared to those obtained from offset locations) demonstrate the value of optimal optode placement, and illustrate the key role mechanical engineering advancements may improve neural data quality.

8.2 Localization: Cap Design

The adjustable localization cap design is based on the customized self-donnable cap design, with added interchangeable shells for each of the three offset positions for the left and right sides (Figure S25). There are three possible configurations for the localization cap, which pairs the left and right position together. Paired offset locations were chosen based on physical compatibility. Three swappable shells encompass locations with a 25 mm offset from the center to the anterior (front), posterior (back), and inferior (down) directions with a fourth shell in the center position. The three offset shells are: *rpla* (right posterior, left anterior), *rilp* (right inferior, left anterior), *rali* (right anterior, left inferior).

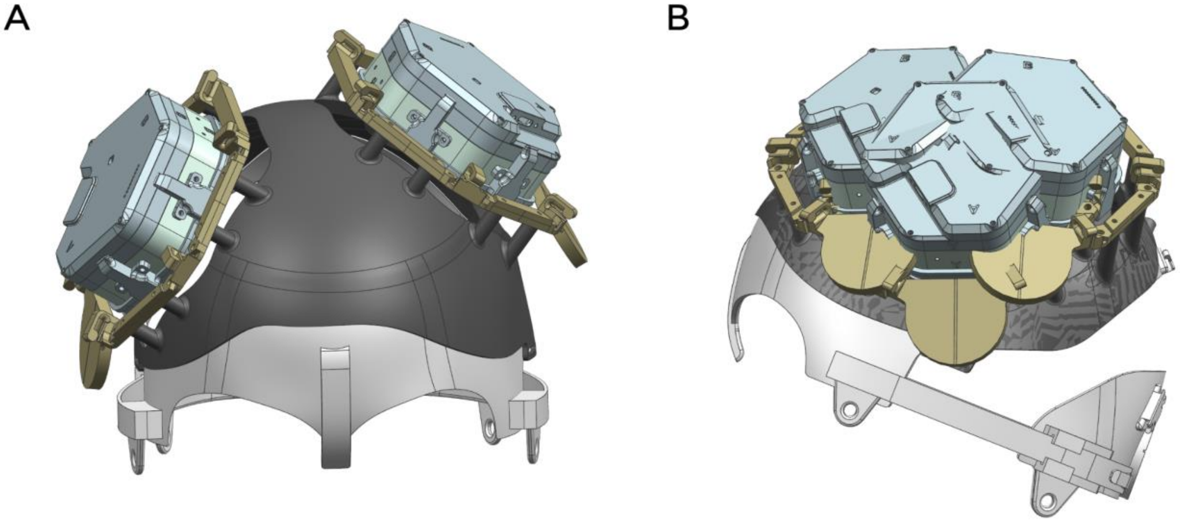

Figure S25. Hardware design of an adjustable localization cap used to evaluate the effect of modules placed off-center: A.) A localization cap showing the modules in the right inferior and left posterior positions. Interchangeable shells (dark gray) enable different offset locations to be tested. B.) Left side view of all three locations overlaid. Modules are offset from the center region in the anterior, posterior, and inferior directions.

8.3 Localization: Experiment protocol

The Localization Experiment is based off the Hemispheric Laterality Experiment with a few minor changes (Figure S19 and S26). When the stimulus displayed a hand cue, participants tapped their thumb, index, and little finger sequentially until the cue disappeared (6 second duration only). Participants remained at rest during the post-stimulus interval (PSI) period (19-21 seconds in duration). There was no null condition in the interest of experiment length. There were 2 conditions: right hand and left hand. Each experiment session consisted of 3 mini-sessions, with each mini session corresponding to a different module offset (*rilp, rali, rpla*). Three sessions were planned in total, to balance the order of module offset. For each mini session (location), 2 blocks were conducted, resulting in 12 trials per condition. Each block was preceded and followed by 20 additional seconds of rest. Experiment duration was roughly 1 hour long (including location changes). All participants underwent a cap-fitting procedure before each location.

Two participants participated in this study, all of whom also participated in the Hemispheric Laterality Experiment. One participant’s data were excluded due to significant differences in SNR between locations (Figure S29).

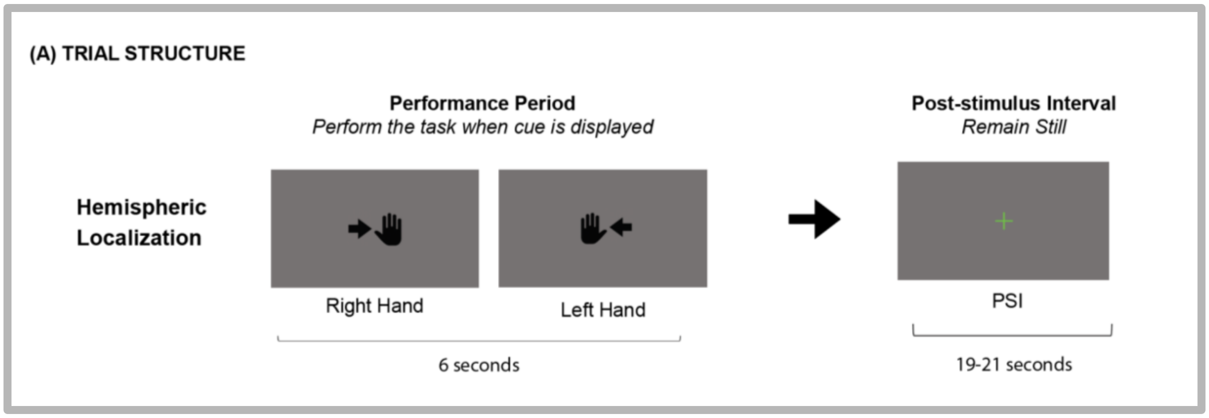

Figure S26. Illustration of the trial structure of the Hemispheric Localization Experiment: six seconds of performance period while visual cue being displayed, followed by 19-21-second post-stimulus internal. Twelve trials per condition were collected in each scalp location, for three locations total. Experiment duration was roughly 1 hour long (including location changes).

8.4 Localization: Results

The ability to measure from the hand-motor cortex, as quantified by the number of significant channels for the LEFT minus RIGHT contrast (Figure S27), is significantly higher when the optode array is centered over the region of interest. Since signals in the hand-motor cortex are large and widespread in the finger-tapping task, even module offsets of 25 mm affords the detection of significant channels -- but note that the number of significant channels is reduced (Figure S28).

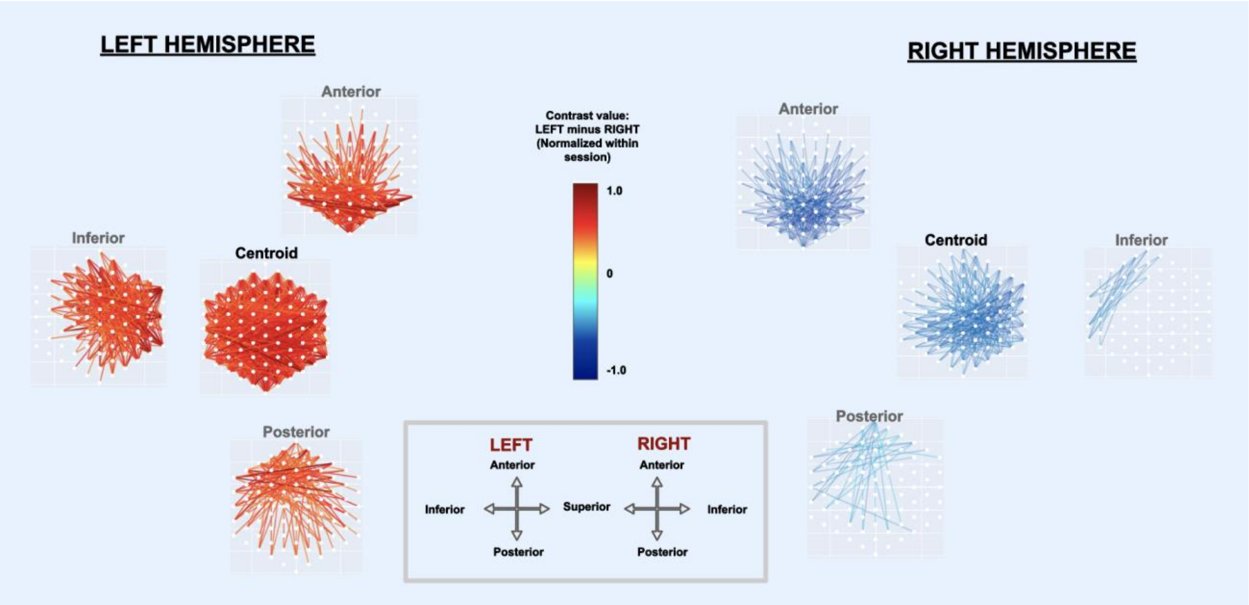

Figure S27. Haystack Plots from Localization Experiment. Lines pertain to the source-detector (SD) pair. Line color indicates the t-stat statistic for the LEFT minus RIGHT GLM contrast (normalized for each session) for the HBR species. Only SD pairs surpassing the 50% threshold (positive and negative) are shown. The middle haystack plot in each hemisphere corresponds to the optode array directly placed over the centroid. The surrounding haystack plots correspond to the respective offset.

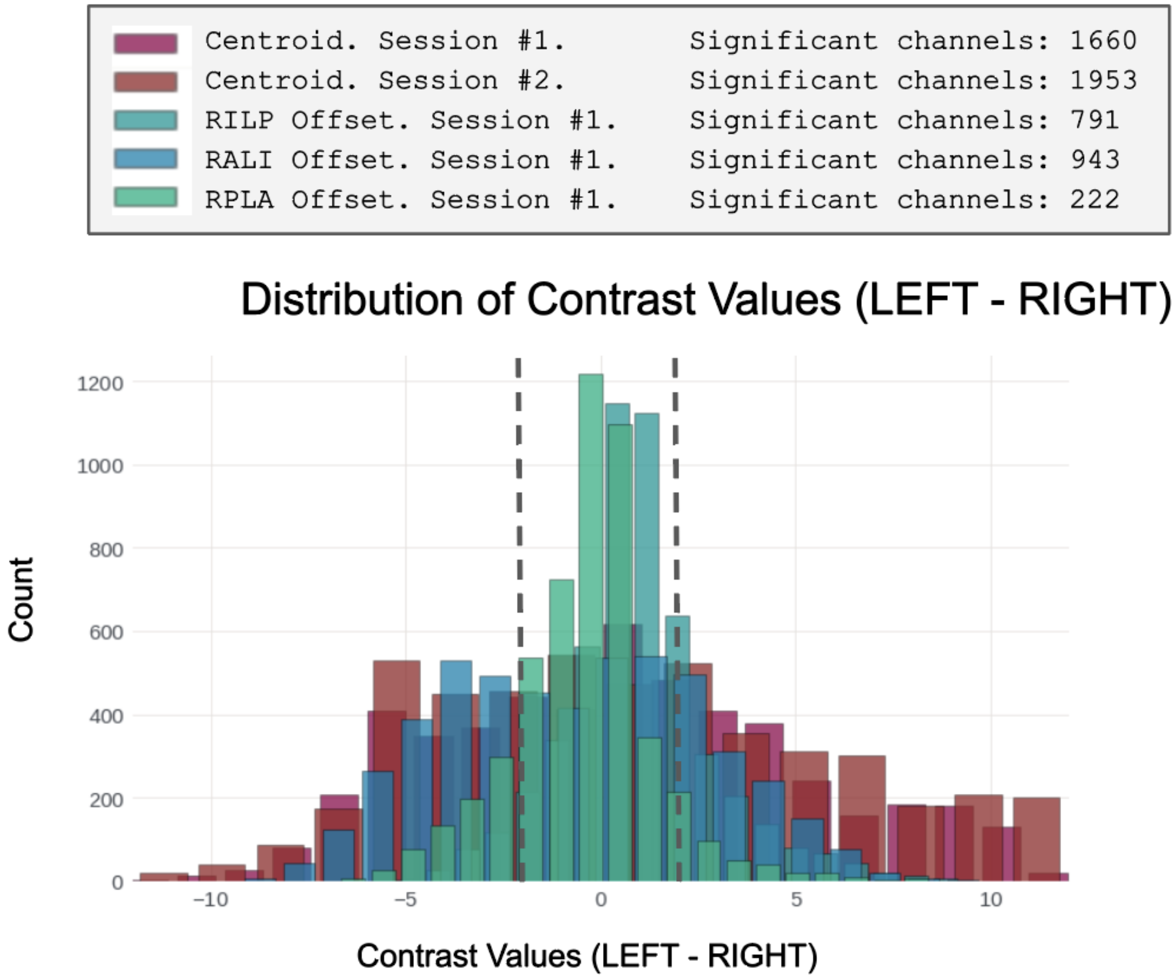

Figure S28. Distribution of Contrast Values. The histogram shows the distributions of t-stats for the Left minus Right GLM contrast (i.e. how well a channel was able to discriminate left versus right hand tapping, with higher absolute value indicating greater significance) for participant MST704. Dashed line indicates the statistical threshold above which the contrast was deemed significant (t > 2 with false discovery rate correction). The legend indicates

the location of the optode array and the number of significant channels that were able to significantly discriminate between left and right hand tapping.

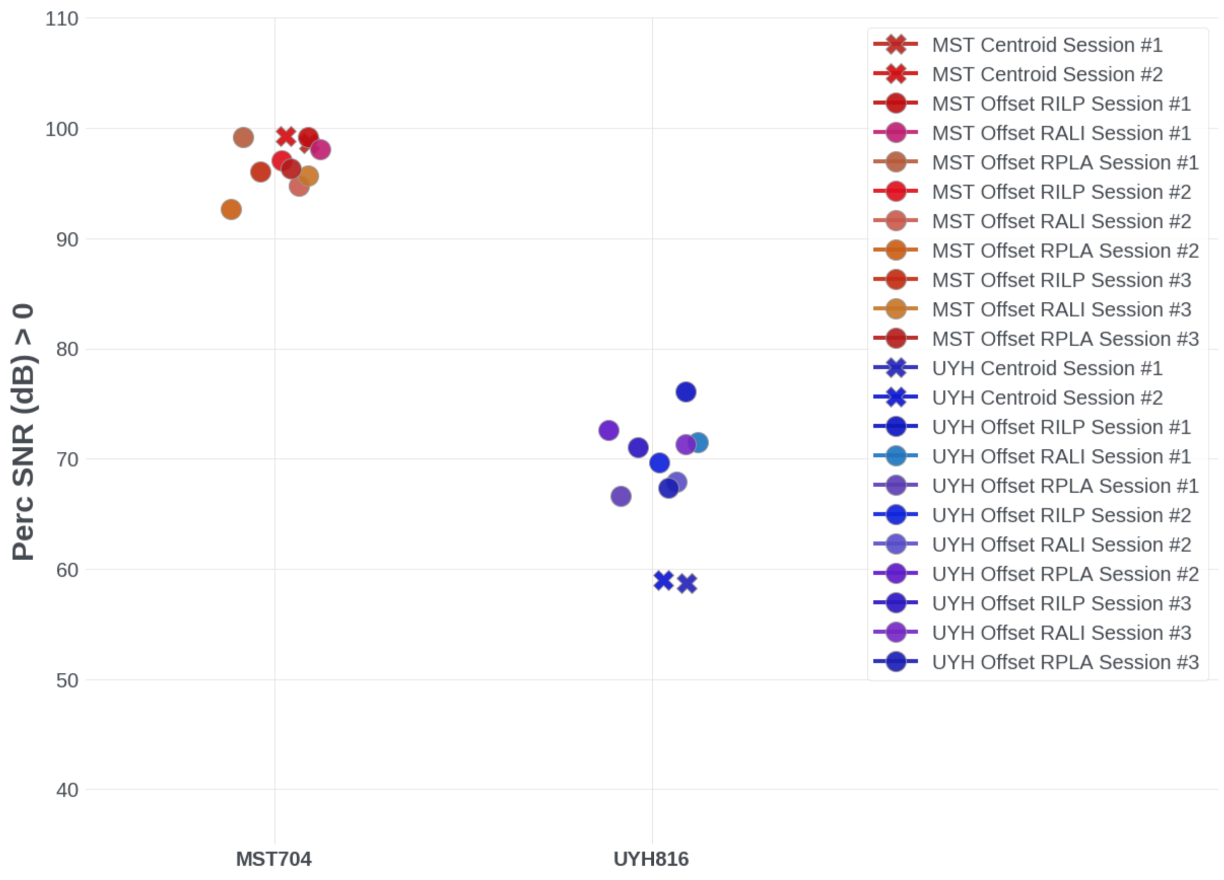

Figure S29. Pulse SNR values across sessions and participants. The pulse SNR metric is the percent of channels where pulse SNR values are greater than 0 (dB). Participant MST704 is shown in red; participant UYH816 is shown in blues. Sessions where the optode array is in the centroid location is indicated with an ‘x’; sessions with offset locations are indicated with circles.

9. LabGraph

LabGraph is a streaming open-source framework built by the Meta Reality Labs Research team. It is particularly well-suited to real-time neuroscience, physiology, and psychology experiments. Spotlight' project uses the LabGraph framework to develop device node integration, closed-loop decoding, and experimental protocols. LabGraph can be assessed from https://github.com/facebookresearch/labgraph
